# Binding selectivity-dependent molecular mechanism of inhibitors towards CDK2 and CDK6 investigated by multiple short molecular dynamics and free energy landscapes

**DOI:** 10.1080/14756366.2022.2135511

**Published:** 2022-11-07

**Authors:** Lifei Wang, Dan Lu, Yan Wang, Xiaoyan Xu, Peihua Zhong, Zhiyong Yang

**Affiliations:** a School of Science, Shandong Jiaotong University, Jinan, PR China; b Department of Physics, Jiangxi Agricultural University, Nanchang, PR China; c College of Computer Information and Engineering, Jiangxi Agriculture University, Nanchang, PR China

**Keywords:** Cyclin-dependent kinase 2, cyclin-dependent kinase 6, binding selectivity, cross-correlation analysis

## Abstract

Understanding selectivity-dependent molecular mechanism of inhibitors towards CDK2 over CDK6 is prominent for improving drug design towards the CDK family. Multiple short molecular dynamics (MD) simulations combined with MM-GBSA approach are adopted to investigate molecular mechanism on binding selectivity of inhibitors X64, X3A, and 4 AU to CDK2 and CDK6. The RMSF analysis and calculations of molecular surface areas indicate that local structural and global flexibility of CDK6 are stronger than that of CDK2. Based on dynamics cross-correlation maps (DCCMs), motion modes of CDK2 and CDK6 produce difference due to associations of X64, X3A, and 4 AU. The calculated binding free energies (BFEs) demonstrate that the compensation between binding enthalpy and entropy of X64, X34, and 4 AU is a key force driving selectivity of inhibitors towards CDK2 over CDK6. This work provides valuable information for designing highly selective inhibitors towards CDK2 and CDK6 and further promotes identification of efficient anticancer drugs in the future.

## Graphical Abstract

**Figure:**
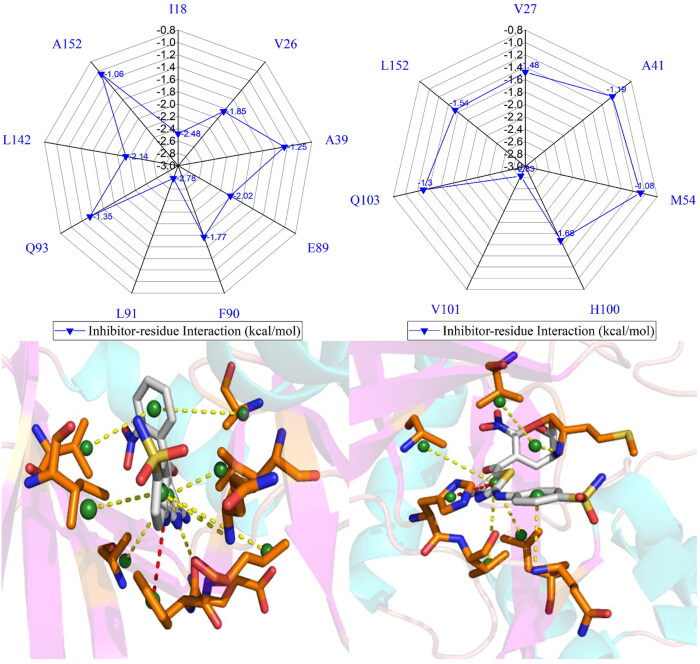

## Introduction

Enhanced resistance to apoptosis and loss of cell-cycle regulation are principal hallmarks of cancers^1^. Cyclin-dependent kinases (CDKs), a family of 13 members containing CDK1-CDK13^2^, are proline-directed serine-threonine kinases which related to an activating cyclin regulatory subunit^3^. CDK2 is an important macromolecule in cell cycle regulation, with taking part in inactivation and phosphorylation of the retinoblastoma protein (RB) tumour suppressor family and regulating both G1/S and G2/M progressions^4^^,^^5^. Furthermore, CDK2 strengthens DNA replication and plays a critical role in cell cycle in the DNA damage response^6^. CDK6 is regarded as a typical cell cycle kinase that boosts arrest of sensitive tumour cells in the G1 phase of the cell cycle. CDK6 takes part in the process of cancer development by using the kinase-dependent or non-kinase-dependent function^7^. Based on importance in promoting cancer initiation as well as progression, CDK2 and CDK6 have drawn intense interest as promising therapeutic targets for cancer.

CDK2 is well known for the critical role that plays in cell cycle progression; concurrently, significantly over-activation of CDK2 is also discovered in neuroblastoma^8^, lung (A549)^9^ cancer, ovarian (SKOV3) cancer^10^ and many other types of cancer^11^. In the past few decades, CDK2 has been regarded as a therapeutic boulevard to restrain cancer cell proliferation, more importantly, many small-molecule inhibitors with various scaffolds have been developted^12^. For instance, CCT068127 designed by Whittaker et al., a novel inhibitor of CDK2, efficiently leads to the decrease of RB phosphorylation, reduction of phosphorylation of RNA polymerase II as well as induction of cell cycle arrest and apoptosis^13^. Wood et al. applied an efficient method to detect interaction sites of inhibitors with proteins, and their findings successfully identifies allosteric and orthosteric sites of CDK2^14^. Apart from diverse experimental works, multiple computational explorations are also used to decode molecular mechanism of inhibitor bindings to CDK2^15–19^. Duan et al. adopted efficient interaction entropy approach and the polarised protein-specific charge force field to decipher the binding modes of five CDK2-ligand complexes, which provides a substantial energy information for design potent and selective inhibitors to CDK2^20^. Wang et al. employed free energy perturbation/replica exchange with solute tempering (FEP/REST) approach to evaluate the binding affinity of various inhibitors to CDK2 and the results indicate that the novel FEP/REST approach maintains consistency and reliability of calculations^21^.

**Figure 1.**
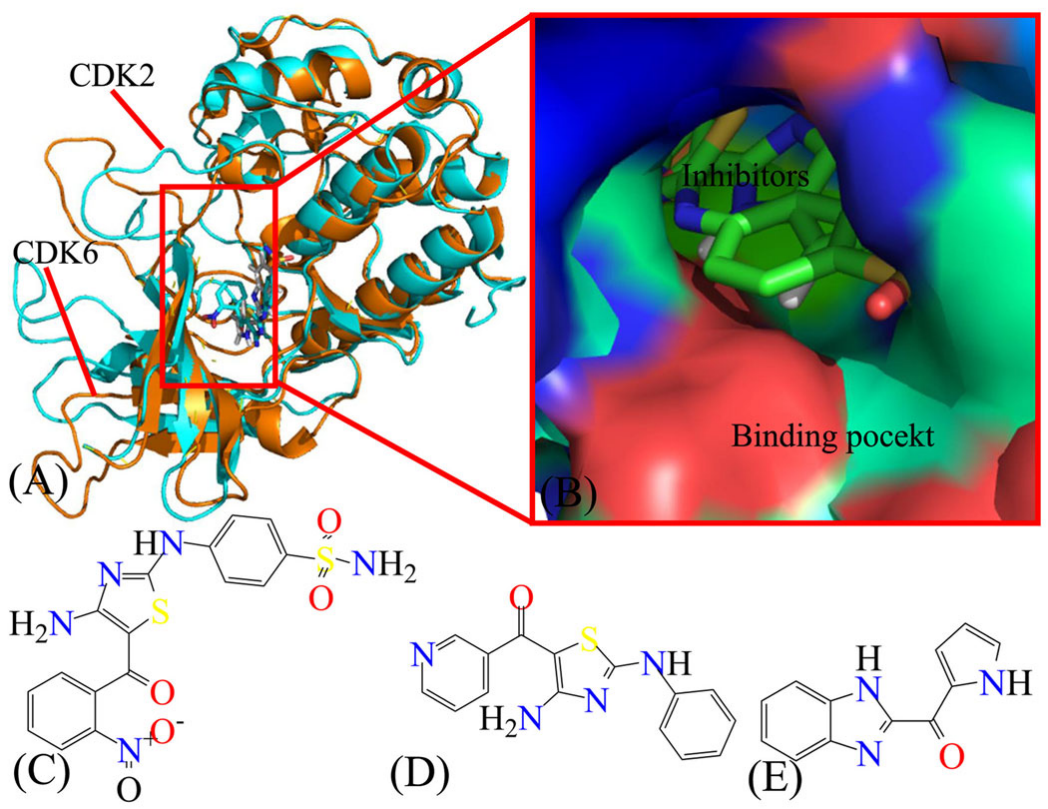
Molecular structures of CDK2/CDK6 and three inhibitors: (A) the structure of cyclin-dependent kinases CDK2 and CDK6 are coloured in cyan and orange, respectively; (B) binding pocket of two different inhibitors to CDK2 and CDK6, among which inhibitors are displayed in stick modes and CDK2 and CDK6 in surface modes; (C–E) separately correspond to the structures of X64, X3A, and 4AU, in which inhibitors are displayed in line modes. In this figure, the crystal structures, PDB code 4GCJ, and 4AUA are applied to respectively represent the structures of the X64-CDK2 and 4AU-CDK6 complexes.

CDK6 is a homologue of CDK2, highly similar sequences and structural topology with CDK2 (Figure 1(A)) that regulates cell cycle progression, which plays an important role in the progression of various types of cancer^22^. Moreover, CDK6 expression is evidently increased in MPN/myelofibrosis haematopoietic progenitor cells and its overexpression is related to diabetes, inflammatory diseases, and cancers^23^. Iris et al. found that CDK6 can enhance cytoskeletal stability of erythroid cells through regulating the transcription of a panel of genes associated with actin (de-) polymerisation^24^. The study from Schmalzbauer et al. indicated that the BSJ-mediated degradation can protect the CDK6-p16(INK4A)/p18(INK4C) complexes and that INK4 levels define the proliferative response to the degradation of CDK6, which reveals that INK4 proteins can be used as predictive signals for CDK6-targeted therapy in acute myeloid leukaemia^25^. Except for diverse experimental works, Yousuf et al. combined molecular docking, fluorescence-based binding, and enzyme inhibition to screen out the best possible inhibitor of CDK6, and they identified quercetin as a potent inhibitor of CDK6, which provides a new avenue for the development of anticancer drugs associated with CDK6 inhibitors^26^. Many works have drawn intense interest on the development of effectively potent and selective inhibitors towards different CDKs^27–29^. For instance, Schonbrunn et al. probed 103,000 complexes derived from commercial library and discovered a panel of 339 kinases with highly binding preference for CDK2 and CDK5 over CDK1, CDK4, CDK6, and CDK9^30^. Besides, pharmacological inhibitors of CDK4 and CDK6 have been regarded as standard treatment among the patients with hormone receptor-positive late staged breast cancer^31^. Dutta et al. investigated the efficacy of CDK4 and CDK6 inhibitor palbociclib alone or in integrating with ruxolitinib in Jak2V617F and MPLW515L murine models of myelofibrosis, and their findings indicate that CDK6 inhibitor palbociclib in integrating with ruxolitinib effectively ameliorates myelofibrosis^23^. Therefore, the atomic-level exploration on the molecular mechanism and conformational variations of CDK2 and CDK6 caused by inhibitor bindings can supply meaningful information for designing highly potent and selective inhibitors towards CDKs.

Conventional molecular dynamics (cMD) simulations^32–38^ and computations of binding free energies (BFEs)^39–43^, including thermodynamics integration^44^^,^^45^, FEP^46^, and solvation interaction energy^47^^,^^48^, etc., have been essential tools for deciphering ligand-protein and small molecule-solvent interactions; moreover, many works have successfully probed the binding selectivity of inhibitors to homologous proteins with similar structural topology and high sequence identity^49–55^. However, the conformations sampled by cMD simulations are possibly trapped in the minimal space, which leads to insufficient conformational sampling. As a matter of fact, multiple short MD (MSMD) simulations can provide more reliable sampling effect than those acquired from a single long MD simulation^56–59^. Thus, MSMD simulations were employed to improve sampling efficiency on CDK2 and CDK6 to investigate molecular mechanism affecting binding selectivity of inhibitors to CDK2 and CDK6. With our expectation, three inhibitors X64, X3A, and 4 AU were selected to explore their selective mechanism on CDK2 and CDK6. Molecular structure, the binding cavity of CDK2 and CDK6, and the structures of X64, X3A, and 4 AU are depicted in Figure 1(A–E). The inhibiting ability of X64 and X3A on CDK2 are scaled by the IC50 values 20 and 650 nM, while that of 4 AU towards CDK6 is scaled by the IC50 value of 7200 nM^30^^,^^60^. Currently, the experimentally binding data of X64 and X3A to CDK6, and that of 4 AU to CDK2 are not available, which inspires the necessity for further insight into binding selectivity of inhibitors on CDK2 and CDK6. In this work, MSMD simulations, MM-GBSA approach, and principal component analysis (PCA)^61–64^ are combined to decode the molecular mechanism concerning the binding selectivity of inhibitors towards CDK2 and CDK6. Meanwhile, this work is also expected to provide structure–function relationship of the inhibitor-bound CDK2 and CDK6 at atomic levels for design of selective inhibitors towards CDKs.

## Theory and methods

### Construction of simulated systems

The initial configurations of CDK2 complexed with X64 and X3A and CDK6 complexed with 4 AU are got from the protein data bank (PDB): The entry ID 4GCJ and 3QTW for the X64- and X3A-CDK2 complexes and 4AUA for the 4 AU-CDK6 complex, respectively^30^^,^^60^. Since the crystal structures of the 4 AU-CDK2, X64-CDK6, and X3A-CDK6 complexes are unavailable in the PDB, the PyMol software (https://www.pymol.org) is applied to dock X64, X3A, and 4 AU into CDK2 and CDK6 so as to produce the missing complexes by performing structural superimposition using 4GCJ and 4AUA as templates of CDK2 and CDK6, separately. The missing residues in the crystal structures of CDK2 and CDK6 are repaired with the MODELLER program^65^ and Chen et al. use this program to perform similar repairs^15^^,^^66^. The protonated states of residues in CDK2 and CDK6 were determined by using the program PROPKA^67^^,^^68^, and the rational protonation was assigned to each residue. The hydrogen atoms unavailable from the crystal structures are bonded to their corresponding heavy atoms with the tool Leap in Amber 20^69^^,^^70^. The *ff*19SB force field^71^ and TIP3P model^72^ are employed to separately assign the force field parameters to two proteins CDK2 and CDK6 and water molecules, respectively. The configuration of X64, X3A, and 4 AU are optimised at the semi-empirical AM1 level, and subsequently, atomic BCC charges of X64, X3A and 4 AU are yielded through the tool Antechamber in Amber 20. The general amber force field (GAFF) is applied to generate the force field parameters of X64, X3A, and 4 AU^73^. Three chloridion ions (Cl^−^) and one sodium ion (Na^+^) are placed around the inhibitor-bound CDK2 and CDK6 to neutralise the simulated systems in the salt environment of 0.15 M NaCl^74^, respectively. Moreover, octahedral periodic boxes of the TIP3P water model with 12.0 Å buffer along each dimension are utilised to solve the inhibitor-CDK2 or CDK6 complexes, and the number of water molecules is about 13,000.

### Multiple short molecular dynamics simulations

To implement efficient MSMD simulations, the initialised system is optimised using the steepest descent minimisation of 2500 steps and conjugate gradient one of another 2500 steps to relieve high-energy contacts and orientations between atoms. Subsequently, the system undergoes a 2-ns moderate heating process from 0 to 300 K by restraining non-hydrogen atoms of the inhibitor-CDK complex with a constant of 2 kcal/(mol·Å^2^) in the NVT condition. Then, each system is further equilibrated for another 2 ns at the temperature 300 K under NPT condition. Finally, three separate 400-ns cMD simulations are carried out at constant temperature (300 K) and pressure (1 bar) by using periodic boundary conditions (PBCs) and particle mesh Ewald (PME) approach to relax each system^75^^,^^76^. Through our MSMD simulations, all hydrogen-nonhydrogen chemical bonds are restrained with the SHAKE approach^77^. The temperatures of six simulated systems are regulated with the Langevin dynamics with a mild damping coefficient of 2.0 ps^−1^ to stabilise long-time step integrators^78^. A rational cut-off value of 10 Å is utilised to estimate electrostatic interactions with the smooth PME approach, and this cut-off is also used to compute van der Waals interactions simultaneously. In this study, 1.2-μs MSMD simulations containing three-replica simulations of 400-ns are performed on the inhibitor-CDK2/6 complexes. In order to facilitate the post-process analysis, the equilibrated sections from trajectories of three separated replica cMD simulation are connected into a single integrated trajectory (SIT). The program pmemd.cuda stemming from Amber 20 is adopted to run all simulations in this work^79^^,^^80^. PCA and calculations of dynamics cross-correlation maps (DCCMs)^81–84^ are run with the module CPPTRAJ^85^ from Amber 20 to understand conformational alterations of CDK2 and CDK6 and the corresponding details are introduced in our previous works^33^^,^^50^.

### Calculations of binding free energies

The alterations in entropy (ΔH) and enthalpy (−TΔS) are two crucial physical phenomenon accompanying association of ligands with targets. The binding strength of ligands to targets are usually scaled with BFEs expressed at the following forum:

(1) $$\Delta G_{\mathrm{bind}}=\Delta H-T\Delta S$$

MM-GBSA and molecular mechanics Poisson Boltzmann surface area (MM-PBSA) are two powerful approaches to evaluate binding affinities of a large number of ligands to targets^86–89^. Based on reliable evaluation and comparison of Hou’s team^90^^,^^91^ on these two approaches, MM-GBSA is wielded to compute BFEs of X64, X3A, and 4 AU to CDK2/CDK6 using Equation (2):

(2) $$\begin{array}{l} \Delta G_{\mathrm{bind}}=\Delta E_{\mathrm{ele}}+\Delta E_{\mathrm{vdW}}+\Delta G_{gb}+\Delta G_{\mathrm{surf}}-T\Delta S\\ =\Delta G_{\mathrm{pol}}+\Delta G_{\mathrm{hydro}}-T\Delta S \end{array}$$

in which $\Delta G_{\mathrm{pol}}$ represents polar interactions of X64, X3A, and 4 AU with CDK2/CDK6 and it arises from the sum of electrostatic interactions $\Delta E_{\mathrm{ele}}$ and polar solvation-free energy $\Delta G_{gb},$ while $\Delta G_{\mathrm{hydro}}$ is hydrophobic interactions of X64, X3A, and 4 AU with CDK2/CDK6 and this term stems from the sum of van der Waals interactions $\Delta E_{\mathrm{vdW}}$ and nonpolar solvation free energy $\Delta G_{\mathrm{surf}}$ is calculated by using the Generalised Born (GB) model from the study of Onufriev’s group^92^.$\Delta G_{\mathrm{surf}}$ is computed with the empirical equation $\Delta G_{\mathrm{nonpol}}=\gamma \times \Delta \mathrm{SASA}+\beta ,$ in which the parameters γ and ΔSASA individually signify the surface tension and the variance in the solvent-accessible surface areas (SASAs) by the presence of ligands. In this work, the values of 0.0072 kcal/(mol Å^−2^) and 0.0 kcal/mol are assigned to the parameters γ and β, respectively^93^. Finally, the changes in the entropy (−TΔS) accompanying the presence of ligands are computed based on 50 snapshots from the SIT by using the mmpbsa_py_nabnmode program inlayed in Amber 20^94^.

### Free energy landscapes analysis

Free energy landscapes (FELs) are an important tool to explore the conformational alterations of proteins and function^95^^,^^96^. An FEL is a mapping of all possible conformations of a molecular entity^95^^,^^97–99^. To reveal energy bases of CDK2 and CDK6 conformational selectivity, FELs are constructed by using the reaction coordinates saved at the SIT with the following equation:

(3) $$G_{i}=-k_{B}Tln(\frac{N_{i}}{N_{\max}})$$

in which ${\;k}_{B}$ is Boltzmann’s constant, *T* is the temperature of simulation systems and 300 K is set in the current calculations. $N_{i}$ is the population of bin *i* and $N_{\max}$ is the population of the most populated bin. Bins with no population are given an artificial barrier scaled as the lowest probability. For this work, the projections of the SIT on the first two components (PC1 and PC2) are used as the reaction coordinates to build the FELs. Different energy levels are displayed using colour-code modes.

## Results and discussion

### Dynamics equilibrium and structural features of CDK2 and CDK6

In order to get full conformation samplings on CDK2 and CDK6, 1.2-μs MSMD simulations, composed of three separate cMD simulations of 400 ns, are implemented on the X64-, X3A-, and 4 AU-bound CDK2 and CDK6, respectively. Root mean square deviations (RMSDs) of backbone atoms from CDK2 and CDK6 are computed relative to the first structure to measure structural stability of CDK2 and CDK6 (Figure S1). The structural fluctuations of all replicas of the X64-, X3A-, and 4AU-bound CDK2/CDK6 are inclining to reach the equilibrium after 100 ns of simulations. Therefore, the equilibrated parts (100–400 ns) from each replica simulation are connected into a SIT, which facilitates for all computations and post-processing analyses.

**Figure 2.**
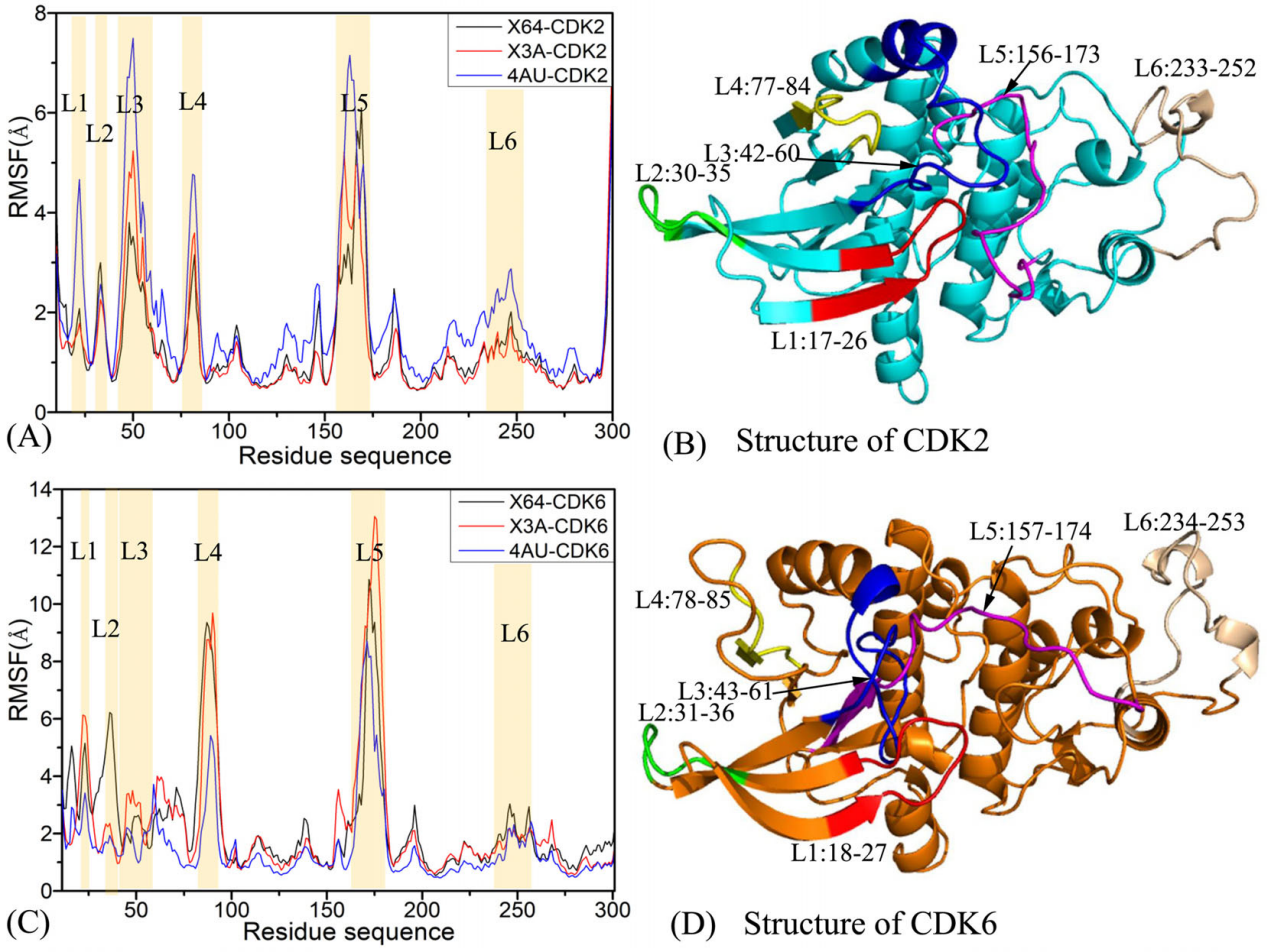
Root-mean-square fluctuations (RMSFs) of the C_α_ atoms in CDK2 and CDK6: (A) RMSFs for CDK2 complexed with inhibitors X64, X3A, and 4 AU, (B) the structure of CDK2, (C) RMSFs for CDK6 complexed with inhibitors X64, X3A, and 4 AU, (D) the structure of CDK6.

To examine the difference in structural flexibilities of CDK2 and CDK6 induced by the presence of X64, X3A, and 4 AU, root mean square fluctuations (RMSFs) of the coordinates of the C_α_ from CDK2 and CDK6 are computed based on the SIT (Figure 2). CDK2 and CDK6 share similar fluctuation tendency of the RMSFs, implying that CDK2 and CDK6 possess common flexible and rigid regions. The large differences in the RMSFs are found in six domains, involved in L1 (residues 17–26 for CDK2 and 18–29 for CDK6), L2 (residues 30–35 for CDK2 and 31–36 for CDK6), L3 (residues 42–60 for CDK2 and 43–61 for CDK6), L4 (residues 77–84 for CDK2 and 78–85 for CDK6, L5 (residues 156–173 for CDK2 and 157–174 for CDK6), and L6 (residues 233–252 for CDK2 and 234–253 for CDK6). Hence some residues existing in the above six domains are possible hot spots of inhibitor-CDKs associations. Based on the comparison of Figure 2(A,C), the RMSF values of most parts from five regions L1, L2, L4, L5, and L6 of CDK6 are obviously bigger than that in the corresponding domains in CDK2, particularly for the regions L4 and L5, demonstrating that local flexibility of CDK6 is stronger than that of CDK2. On the contrary, the RMSF values the in region L3 of CDK6 are lower than that in the corresponding region of CDK2, verifying that the structural flexibility of L3 in CDK6 is weaker than that in CDK2. Structurally, the regions L1 and L2 are near the binding pocket of CDK2 and CDK6, implying that some residues from the secondary structure L1 and L2 primarily promote binding selectivity of X64, X3A, and 4 AU to CDK2 and CDK6. Although four regions L3, L4, L5, and L6 are not in the vicinity of the inhibitor-CDK binding pocket, their fluctuations in structural flexibility can exert considerably impacts on binding of X64, X3A, and 4AU to CDK2 and CDK6.

**Figure 3.**
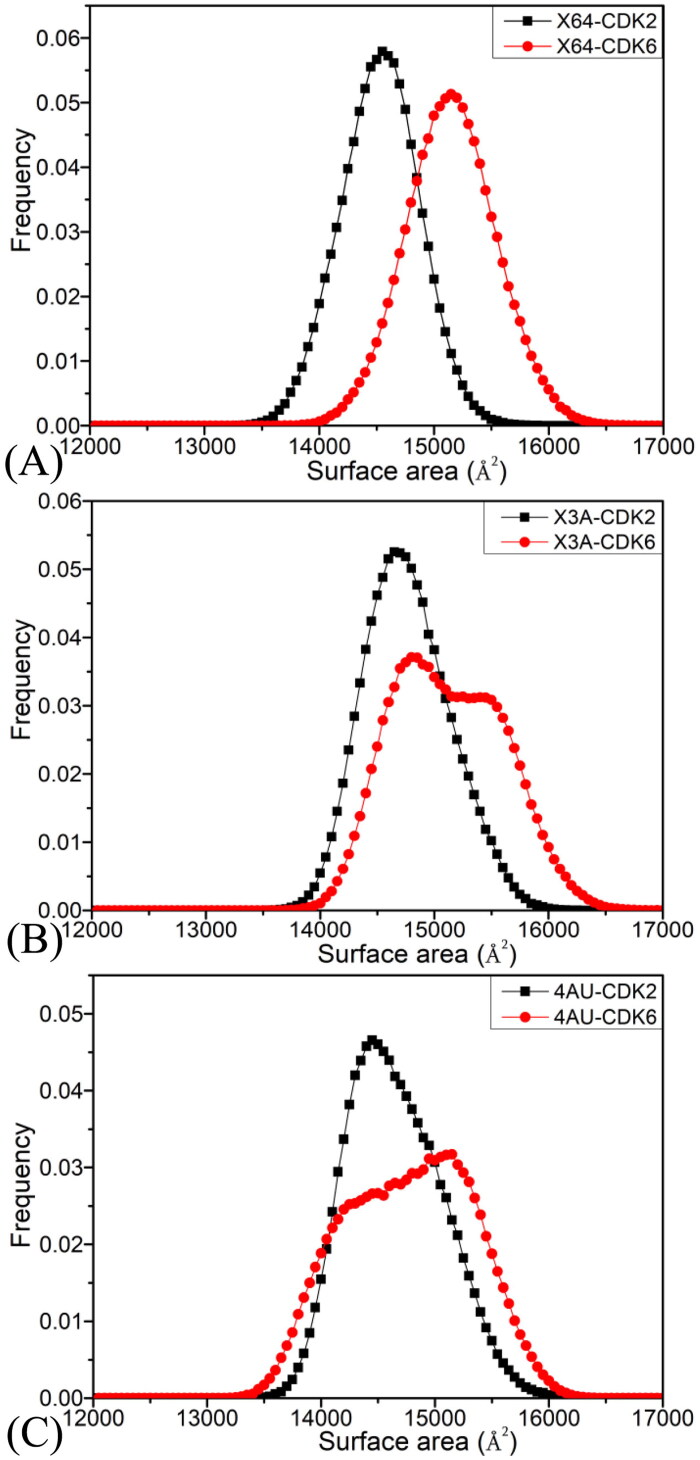
Frequency distribution of the MSAs of the inhibitor-CDK2/CDK6 complexes: (A) the X64-CDK2 and X64-CDK6 complexes; (B) the X3A-CDK2 and X3A-CDK6 complexes; (C) the 4AU-CDK2 and 4 AU-CDK6 complexes.

Molecular surface areas (MSAs) are usually utilised to reflect global structural flexibility of targets. The MSAs of CDK2 and CDK6 are estimated with the SIT and their frequency distribution are depicted in Figure 3. The peak values of the MSA frequencies of the X64-, X3A-, and 4AU-bound CDK2 are primarily located at 14570, 14698, and 14453 Å,^2^ separately, while that of X64-, X3A-, and 4 AU bound-CDK6 are mainly situated at 15161, 14856, and 15141 Å,^2^ separately, showing that global structural flexibility of the inhibitor-bound CDK6 is stronger than that of the inhibitor-bound CDK2. This difference can produce certain influences on binding of X64, X3A, and 4AU to CDK2 and CDK6.

### Internal dynamics of CDK2 and CDK6

**Figure 4.**
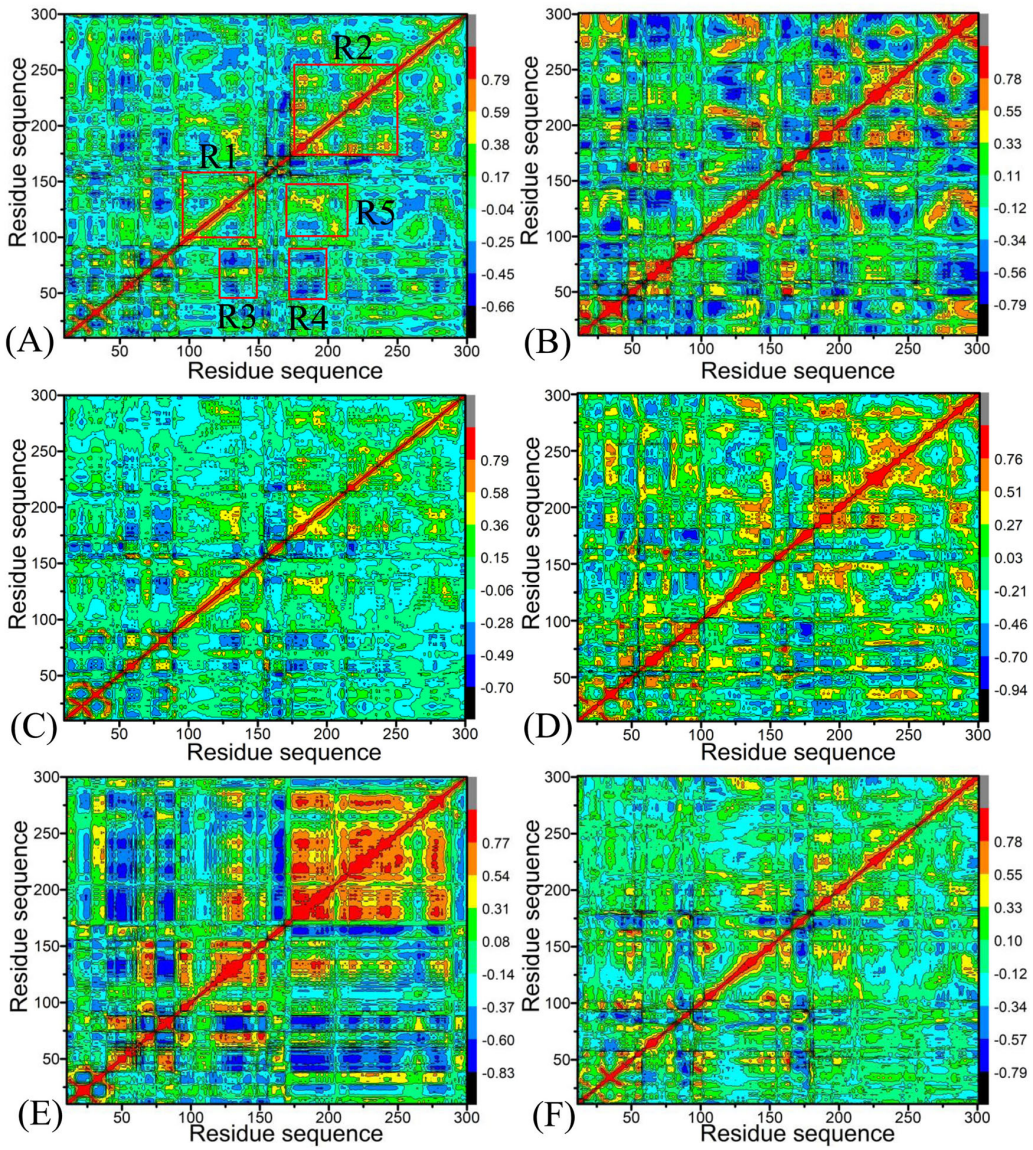
DCCMs calculated by using the coordinates of the $C_{\alpha }$ atoms around their mean positions from the SIT: (A), (C), and (E) corresponding to CDK2 complexed with X64, X3A, and 4AU, separately and (B), (D), and (F) corresponding to CDK6 complexed with X64, X3A, and 4AU, respectively.

To unveil the difference in the internal dynamics behaviour (IDB) of CDK2 and CDK6 caused by binding of X64, X3A, and 4AU to CDK2 and CDK6, DCCMs are calculated with the C_α_ atomic coordinates saved in the SIT (Figure 4). The yellow and red indicate strongly positive correlated (PC) motions, while the dark blue or blue represent strongly anti-correlated (AC) movements. The off-diagonal regions characterise the relative motions between the different residues, while the diagonal ones signify the motion of a certain residue relative to itself. As shown in Figure 4, binding of X64, X3A, and 4AU generates obvious influences on IDB of CDK2 and CDK6.

For CDK2 associated by X64, X3A, and 4AU (Figure 4(A,C,E)), the diagonal regions R1 and R2, as well as the off-diagonal R5 region, produce strongly PC motions, but the regions R3 and R4 generate significantly AC motions.

As shown in Figure 4(A,B), the presence of X64 in CDK6 not only strengthens the PC movement in the R1, R2, and R5 compared to the X64-bound CDK2, but also slightly heightens the AC motion in the R3 and R4. The binding of X3A to CDK6 also enhances the PC movements in the R1, R2, and R5 relative to the X3A-bound CDK2, as well as heightens the AC motions in the R3 and R4 (Figure 4(C,D)). The association of 4AU with CDK6 not only evidently weaken the PC motions in the R1, R2, and R5 by referencing the 4AU-bound CDK2 but also decreased the AC movements in the R3 and R4 (Figure 4(E,F)). On the basis of the current analysis, associations of identical inhibitors yield evident differences in the IDBs between CDK2 and CDK6, verifying that certain residues situated in the R1–R5 may play a pivotal role in binding selectivity of X64, X3A, and 4AU to CDK2 over CDK6.

PCA is extensively employed investigate concerted motions (CMs) of structural domains from targets. PCA can be realised through a diagonalisation on a covariance matrix constructed with the $C_{\alpha }$ atomics coordinates recorded in the SIT (Figure S2). The first six principal components of CDK2 bound by X64, X3A, and 4AU, describing momentously collected motions, occupy 79.40%, 76.74%, and 68.52% of the observed motions in MSMD simulations, respectively, while that of CDK6 complexed with X64, X3A, and 4AU account for 71.48%, 72.90%, and 70.13% of the total motions from the MSMD simulations, separately. The first six eigenvalues of the X64-, X3A, and 4AU-bound CDK6 are decreased relative to the one of the X64-, X3A, and 4AU-associated CDK2, showing that the presence of X64, X3A, and 4AU in CDK2 and CDK6 produces vital influences on the total movement strength of CDK2 and CDK6. These results imply that the difference in IDBs of CDK2 and CDK6 plays a key role in selectivity of X64, X3A, and 4AU towards CDK2 and CDK6.

### Conformational changes of inhibitors to CDK2 and CDK6 probed by free energy landscapes

**Figure 5.**
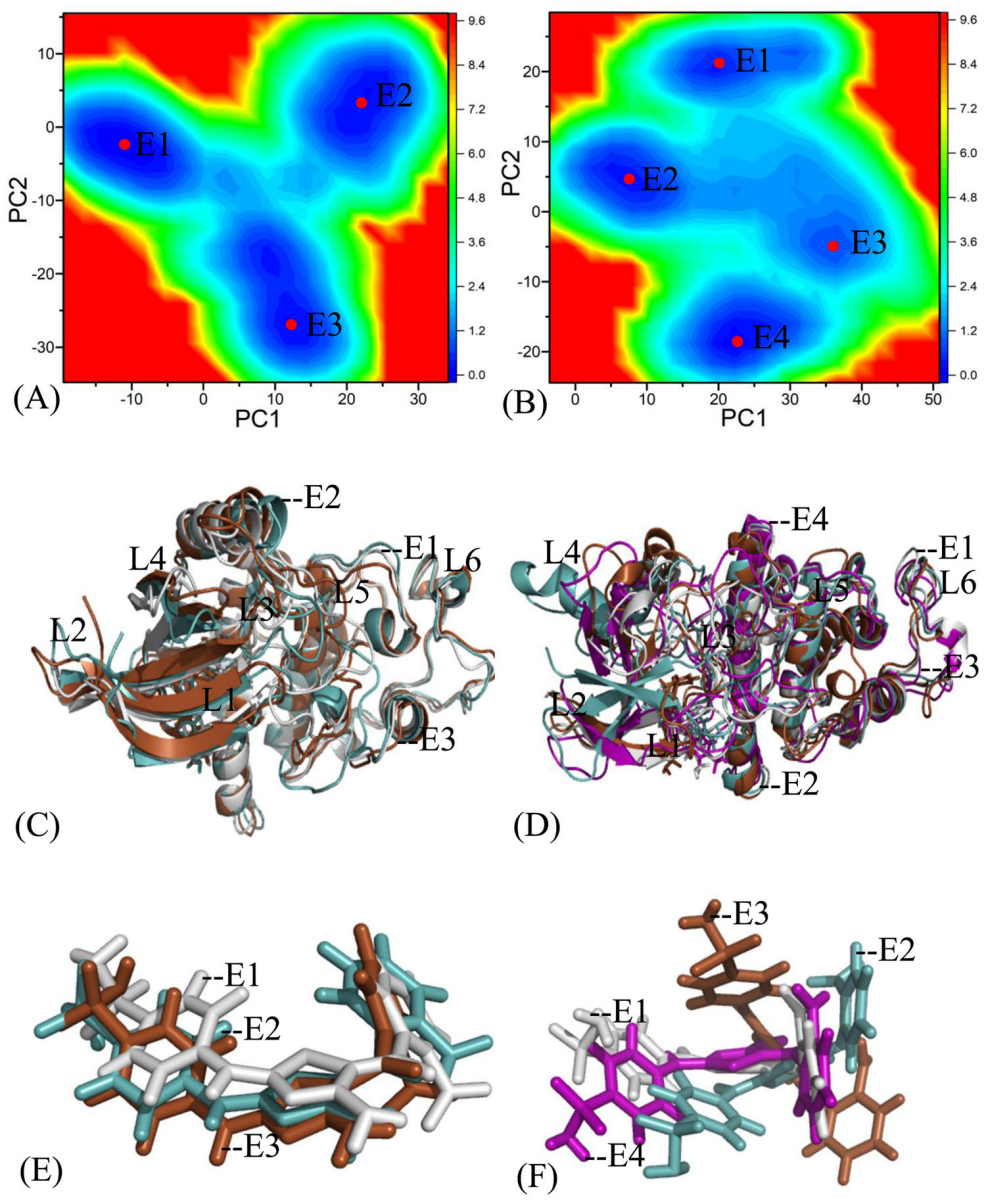
Free energy landscapes and structural information: (A) and (B) separately corresponding to free energy landscapes of the X64-bound CDK2 and CDK6; (C) and (D), respectively, indicating structural superimpositions of the X64-bound CDK2 and CDK6 situated at different potential wells; (E) and (F) separately indicating structural alignments of X64 in different energy wells. CDK2 and CDK6 are displayed in cartoon and X64 in stick modes.

**Figure 6.**
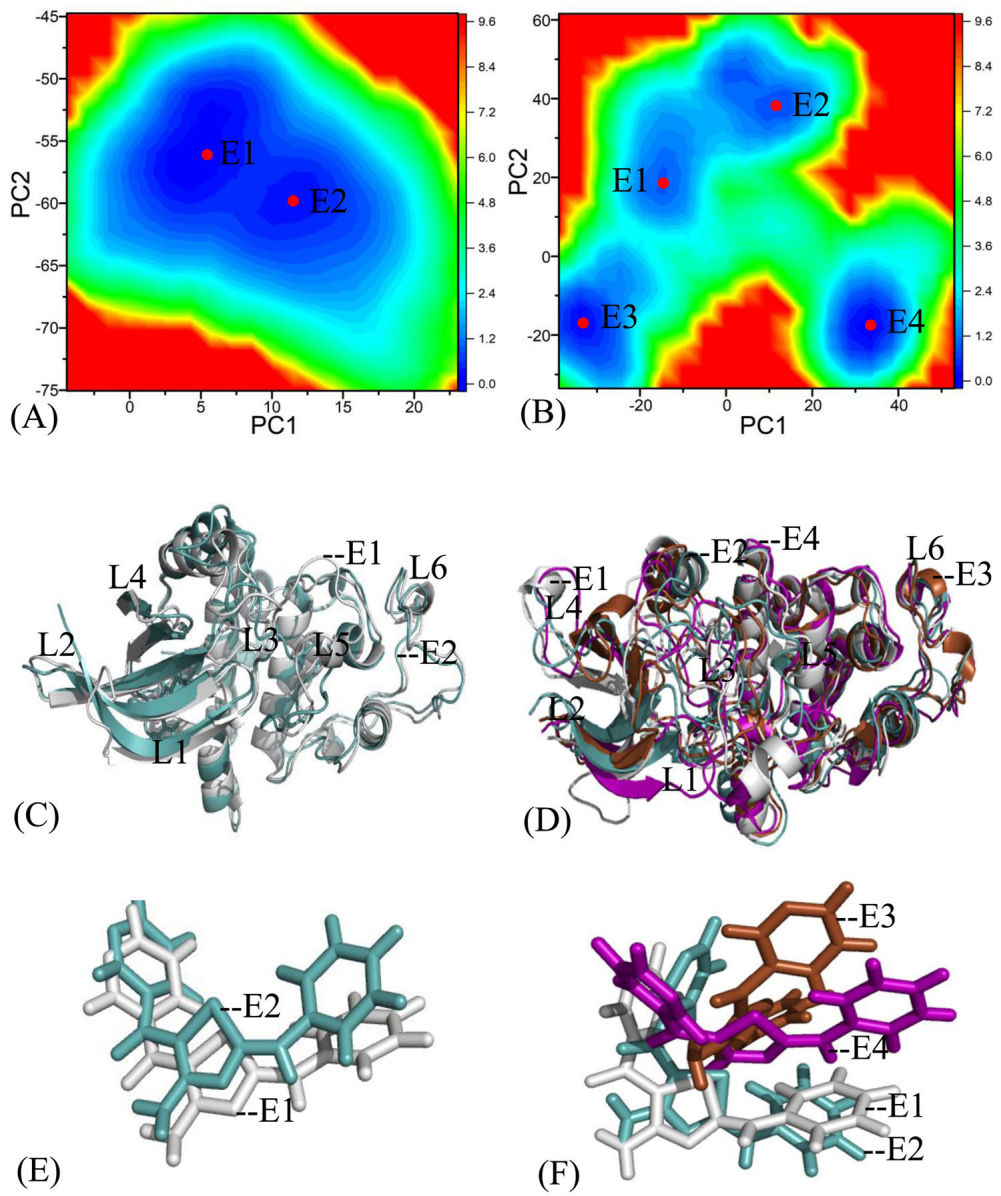
Free energy landscapes and structural information: (A) and (B) separately corresponding to free energy landscapes of the X3A-bound CDK2 and CDK6; (C) and (D), respectively, indicating structural superimpositions of the X3A-bound CDK2 and CDK6 situated at different energy wells; (E) and (F) separately indicating structural alignments of X3A in different energy wells. CDK2 and CDK6 are displayed in cartoon styles and X3A in stick modes, respectively.

**Figure 7.**
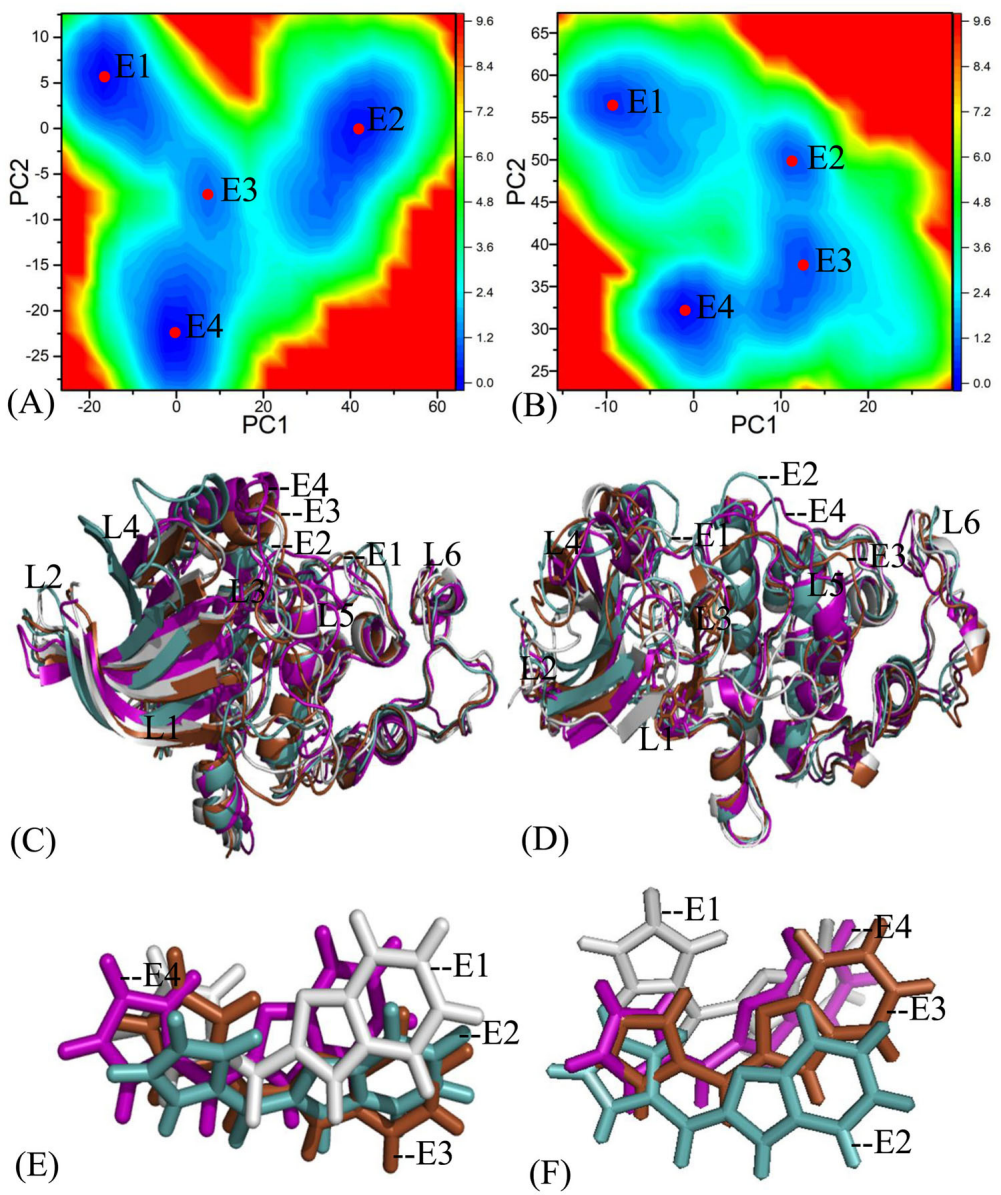
Free energy landscapes and structural information: (A) and (B) separately corresponding to free energy landscapes of the 4AU-bound CDK2 and CDK6; (C) and (D) respectively indicating structural superimpositions of the 4AU-bound CDK2 and CDK6 situated at different energy wells; (E) and (F) separately indicating structural alignments of 4AU at different energy wells. CDK2 and CDK6 and 4AU are displayed in cartoon patterns and 4AU in stick modes, respectively.

FEL is an effective approach to investigate conformational alterations of targets induced by changes of binding environment^100^. To probe conformational changes of CDK2 and CDK6 due to inhibitor bindings, projections of the SIT onto the first two eigenvectors (PC1 and PC2) got from PCA are used as reaction coordinates to yield FELs of CDK2 and CDK6 (Figures 5–7 and Figure S3). From these figures, the symbols E1, E2, E3, E4, and red dots indicate diverse energy wells identified by MSMD simulations. As shown in Figures 5–7 and S3, binding of three inhibitors X64, X3A, and 4AU to CDK2 and CDK6 obviously changes the FELs and produces the conformational rearrangement.

The X64-bound CDK2 *versus* the X64-bound CDK6: MSMD simulations detect three main energy wells E1, E2, and E3 in the X64-associated CDK2 (Figure 5(A)) and these energy wells have almost same depth, demonstrating that the conformations of the X64-CDK2 complex are primarily distributed in three conformational subspaces. Three representative structures situated at the E1, E2, and E3 are superimposed at Figure 5(C) and the information indicates that the domains L3 and L5 evidently deviate from each other, implying that these two secondary structures display a large structural flexibility and play a pivotal role in the function of the X64-CDK2 complex. As shown in structural alignment of X64 in the representative structures E1, E2, and E3 (Figure 5(E)), X64 only produces slight torsion or sliding. For the X64-CDK6 complex, four energy wells E1, E2, E3, and E4 are identified through the entire MSMD simulation energy wells (Figure 5(B)), illustrating that the X64-CDK6 complex is populated four conformational subspaces. The alignments of four representative structures situated at the energy wells E1, E2, E3, and E4 indicate that the domains L1, L3, L4, and L5 generate obvious deviations from each other (Figure 5(D)). The active domains L1, L3, L4, and L5 generate significant torsion among four representative structures, which possibly bring obvious influences on the binding of X64 to CDK6, which is supported by the obvious structural deviations of X64 revealed by the structural superimposition of X64 (Figure 5(F)). Therefore, the conformational changes of L3 and L5 and torsion of X64 must yield obvious impacts on binding selectivity of X64 to CDK2 and CDK6.

The X3A-associated CDK2 over the X3A-bound CDK6: two energy wells E1 and E2 emerge at the MSMD simulations of the X3A-associated CDK2 and in the light of the colour bar two energy wells have almost identical depth (Figure 6(A)), reflecting that the X3A-CDK2 complex spans two major conformation subspaces. The superimpositions of two typical structures situated at the energy wells E1 and E2 suggest that the domain L3 has bigger structural flexibility and evidently deviate from each other in the X3A-bound CDK2 (Figure 6(C)). The structures of X3A in two typical structures E1 and E2 are superimposed together (Figure 6(E)) and the results demonstrate that X3A produces evident slide, which implies an evident effects on binding of X3A to CDK2. For the X3A-boundCDK6, four energy wells E1, E2, E3, and E4 are recognised by the entire MSMD simulation and on the basis of colour bar the depth of the energy wells in the states E3 and E4 are deeper than that of the energy wells E1 and E2 (Figure 6(B)), signifying that the structures of the X3A-CDK6 are clustered into four conformational spaces. The alignments of the X3A-associated CDK6 situated at the energy wells E1, E2, E3, and E4 demonstrate that the domains L1, L3, and L5 generate evident deviations from each other (Figure 6(D)). The structures of X3A in four representative structures of the X3A-bound CDK6 are superimposed together (Figure 6(F)) and the results indicate that X3A has four different binding poses and yield heavy deviations, which extremely affects binding of X3A to CDK6. Thus, the alterations in conformations and orientations definitely affect binding selectivity of X3A to CDK2 and CDK6.

The 4AU-bound CDK2 against the 4 AU-associated CDK6: MSMD simulations capture four energy wells E1, E2, E3, and E4 and according to the colour bar three typical structures E1, E2, and E4 are situated at a deeper potential well than the typical structure E3 (Figure 7(A)), implying that the 4 AU-CDK2 complex possess four main conformations. The superimpositions of four representative structures corresponding to the energy wells E1, E2, E3, and E4 display that the three domains L3, L4, and L5 produce obvious deviations from each other (Figure 7(C)), signifying that these three secondary structures have larger flexibility and play a critical role in the function of the 4 AU-CDK2 complex. The structures of 4AU in four typical conformations E1, E2, E3, and E4 of the 4 AU-bound CDK2 are aligned together (Figure 7(E)). The results reveal that 4 AU has four different binding poses in CDK2, which yields vital impact on binding of 4AU to CDK2. Concerning the 4 AU-associated CDK6 four distinct energy wells E1, E2, E3, and E4 are distinguished through the whole MSMD simulation and the colour bar exhibits the depth of the energy wells E1 and E4 is deeper than that of the energy wells E2 and E3 (Figure 7(B)), illustrating that the structures of the 4 AU-CDK6 complex are mainly distributed at four conformational subspaces. The alignments of four structures situated at the energy wells E1, E2, E3, and E4 indicate that the domains L2, L3, and L4 generate significant deviations from each other (Figure 7(D)), which possibly induce evident influences on binding of 4AU to CDK6. The structural superimpositions of 4AU from four typical structures of the 4 AU-bound CDK6 in the energy wells E1, E2, E3, and E4 signify that the binding poses of 4AU in CDK6 are highly different from each other, which exerts certain effect on association of 4AU with CDK6.

### Binding affinity of inhibitors to CDK2 and CDK6

To decipher binding difference of X64, X3A, and 4 AU to CDK2 and CDK6, MM-GBSA approach is employed to compute BFEs of them to CDK2 and CDK6 by using 300 structural frames extracted from the 900-ns SIT with a time interval of 3 ns. In order to avoid expensive computational time, only 50 snapshots chosen from the above 300 structural frames are used to perform calculations of the entropy contributions to binding of X64, X3A, and 4AU (Table 1). It is encouraging that the rank of our estimated BFEs is consistent with that of available experimental values, implying that the energetic information revealed by BFEs is reliable.

**Table 1.** Binding affinities of inhibitors X64, X3A and 4 AU to CDK2 and CDK6 computed with the MM-GBSA approach^a^.

Terms	X64-CDK2		X64-CDK6		X3A-CDK2		X3A-CDK6		4AU-CDK2		4AU-CDK6	
	Mean	^b^Sem	Mean	^b^Sem	Mean	^b^Sem	Mean	^b^Sem	Mean	^b^Sem	Mean	^b^Sem
$\Delta E_{\mathrm{ele}}$	−40.56	0.56	−44.97	1.11	−29.09	0.30	−13.13	0.47	−17.61	0.28	−16.93	0.52
$\Delta E_{\mathrm{vdW}}$	−48.06	0.14	−36.73	0.75	−38.10	0.22	−29.84	0.93	−26.15	0.34	−22.63	0.50
$\Delta G_{gb}$	53.98	0.39	54.58	1.19	38.05	0.24	25.22	0.73	24.47	0.29	25.36	0.61
$\Delta G_{\mathrm{surf}}$	−4.48	0.02	−3.43	0.07	−3.51	0.02	−2.51	0.08	−2.39	0.03	−2.08	0.05
^c^ $\Delta G_{\mathrm{pol}}$	13.42	0.47	9.61	1.15	8.96	0.27	12.09	0.60	6.86	0.28	8.43	0.57
^d^ $\Delta G_{\mathrm{hydro}}$	−52.54	0.13	−40.16	0.41	−41.61	0.12	−32.35	0.50	−28.54	0.18	−24.71	0.27
^e^ ΔH	−39.12	0.30	−30.55	0.67	−32.65	0.24	−20.26	0.68	−21.68	0.68	−16.28	0.38
-TΔS	22.28	1.07	20.98	1.37	20.64	1.35	14.69	1.47	14.09	1.47	12.31	0.97
$\Delta G_{\mathrm{bind}}$	−16.84		−9.57		−12.01		−5.57		−7.59		−3.97	
IC_50_(nm)	20		^f^–		650		^f^–		^f^–		7200	
^g^ $\Delta G_{\exp}$	−14.60				−8.46						−7.03	

^a^All components of free energies are in kcal/mol.

^b^Standard errors of means (Sem).

^c^ $\Delta G_{\mathrm{pol}}=\Delta E_{\mathrm{ele}}+\Delta G_{gb}.$

^d^ $\Delta G_{\mathrm{hydro}}=\Delta E_{\mathrm{vdW}}+\Delta G_{\mathrm{surf}}.$

^e^ΔH=$\Delta E_{\mathrm{ele}}+\Delta G_{gb}+\Delta E_{\mathrm{vdW}}+\Delta G_{\mathrm{surf}}.$

^f^The experimental binding data is not available.

^g^The experimental values were generated from the experimental IC_50_ values in reference^30^^,^^60^ using the equation $\Delta G_{\exp}=-RT\ln{\mathrm{IC}}_{50}.$

As suggested in Table 1, favourable electrostatic interactions ($\Delta E_{\mathrm{ele}}$) in the gas phase are completely screened by unfavourable polar solvation-free energies ($\Delta G_{gb}$) to provide unfavourable polar interactions ($\Delta G_{\mathrm{pol}}$) for associations of X64, X3A, and 4 AU with CDK2 and CDK6. The changes of entropies (−TΔS) also unfavourable for bindings of X64, X3A, and 4 AU to CDK2 and CDK6. On the contrary, van der Waals interactions ($\Delta E_{\mathrm{vdW}}$) and nonpolar solvation free energies ($\Delta G_{\mathrm{surf}}$) contribute favourable forces ($\Delta G_{\mathrm{hydro}}$) to associations of X64, X3A, and 4AU with CDK2 and CDK6. The BFEs of X64, X3A, and 4 AU to CDK2 are increased by 7.27, 6.44, and 3.62 kcal/mol compared to those of X64, X3A, and 4AU to CDK6, individually, implying that the binding strength of X64, X3A, and 4AU to CDK2 are stronger than those of them to CDK6. The changes of entropy due to the binding of X64-, X3A-, and 4AU to CDK2 are improved by 1.30, 5.95, and 1.78 kcal/mol, separately, relative to that due to the binding of X64-, X3A-, and 4AU to CDK6, which weakens the associations of inhibitors with CDK2 relative to CDK6. However, the changes of enthalpy (ΔH) induced by the X64-, X3A-, and 4 AU-CDK2 bindings are enhanced by 8.57, 12.39, and 5.40 kcal/mol, individually, compared to the X64-, X3A-, and 4AU-CDK6 bindings, which entirely compensates the unfavourable contributions brought by the increases of entropy in the X64-, X3A-, and 4AU-CDK2 bindings relative to those towards CDK6. Hence, the enhancement in enthalpy induced by inhibitor associations towards CDK2 compared with CDK6 entirely dominate the binding selectivity of inhibitors towards CDK2 over CDK6.

### Bing selectivity deciphered by inhibitor-residue interactions

**Figure 8.**
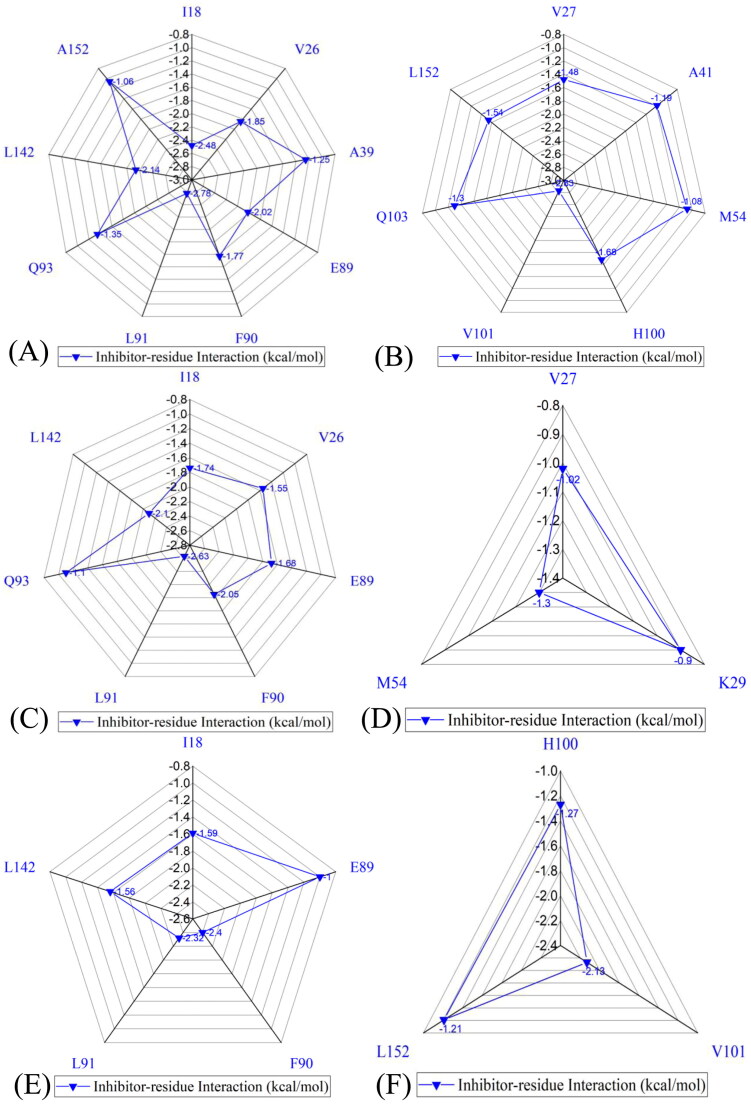
Inhibitor-residue interactions computed by using residue-based free energy decomposition method, only residues stronger than 0.9 kcal/mol are listed: (A) the X64-CDK2 complex, (B) the X64-CDK6 complex, (C) the X3A-CDK2 complex, (D) the X3A-CDK6 complex, (E) the 4AU-CDK2 complex and (F) the 4AU-CDK6 complex.

**Figure 9.**
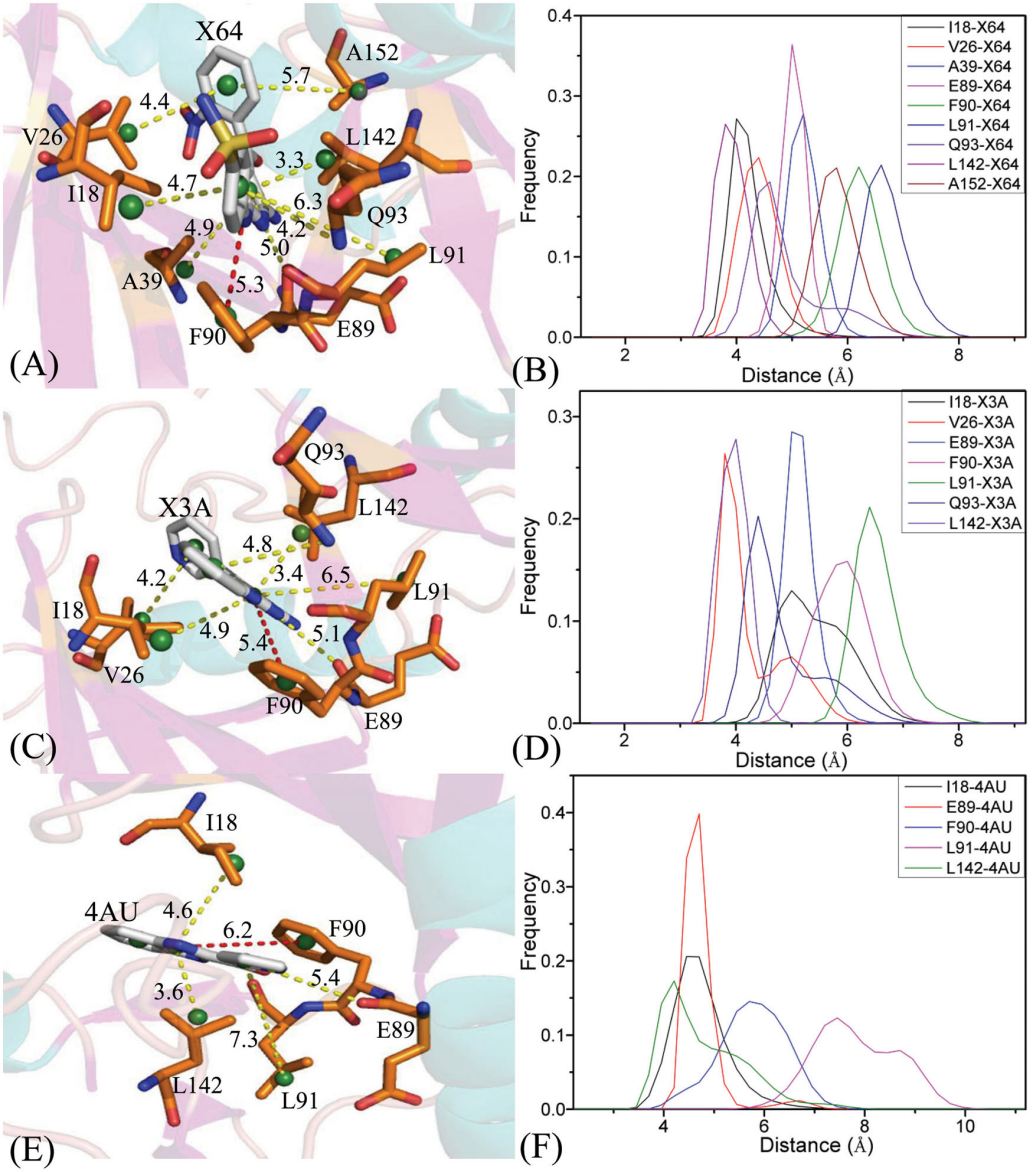
Hydrophobic interactions and frequency distribution of distance involved in interactions of inhibitors with key residues: (A) the X64-CDK2 complex; (B) RDF of X64-CDK2; (C) the X3A-CDK2 complex; (D) RDF of X3A-CDK2; (E) the 4AU-CDK2 complex; (F) RDF of 4AU-CDK2. The frequency of distances between atoms involving significant interactions was calculated by using the integrated MSMD trajectories of the last 900 ns. The yellow dash lines describe the CH–π interactions and the red dash ones indicate the π–π interactions.

**Figure 10.**
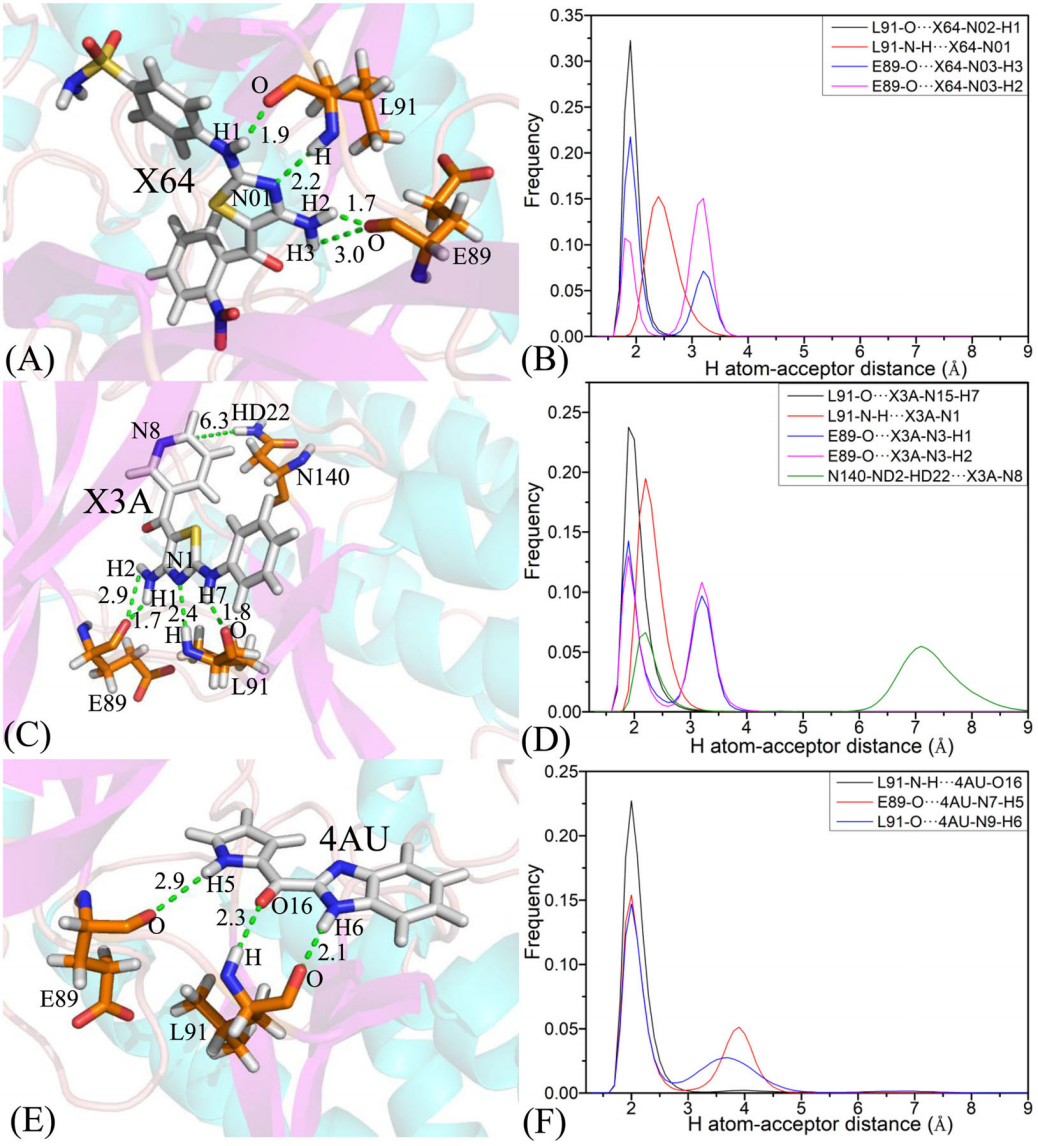
Hydrogen bonds and the corresponding radial distribution function (RDF) of H-O distance between three inhibitors and key residues of CDK2: (A) the X64-CDK2 complex; (B) RDF of H-O distance between L91-O and X64-N02-H1, L91-N-H and X64-N01, E89-O and X64-N03-H3, and E89-O and X64-N03-H2; (C) the X3A-CDK2 complex; (D) RDF of H-O distance between L91-O and X3A-N15-H7, L91-N-H and X3A-N1, E89-O and X3A-N3-H1, E89-O and X3A-N3-H2, and N140-ND2-HD22 and X3A-N8; (E) the 4 AU-CDK2 complex and (F) RDF of H-O distances between L91-N-H and 4 AU-O16, E89-O and 4 AU-N7-H5, and L91-O and 4 AU-N9-H6.

To illuminate binding selectivity of X64, X3A, and 4 AU to CDK2 *versus* CDK6, the contribution of individual residues to the BFEs is estimated with the MM-GBSA approach. Critical residues of CDK2 and CDK6 with energetic contributions bigger than 0.9 kcal/mol are shown in Figures 8 and 9 and S4–S5. Furthermore, the CPPTRAJ program in Amber 20 is used to identify hydrogen bonding interactions (HBIs) of X64, X3A, and 4 AU with CDK2 and CDK6 (Table 2). Hydrogen bonds (HBs) and the corresponding to radial distribution function (RDF) of H-O distance of X64, X3A, and 4 AU away from important residues in CDK2 and CDK6 are depicted in Figures 10 and S6.

**Table 2.** Hydrogen bonding interactions of inhibitors with CDK2 and CDK6.

Complexes	Hydrogen Bonds	Distance/(·Å)^a^	Angle/□^a^	Occupancy/(%)^b^
X64-CDK2 DBRD	L91-O···X64-N02-H1^c^	2.82	154.69	99.54
	L91-N-H···X64-N01	3.30	157.64	81.77
	E89-O···X64-N03-H3	2.78	149.15	67.80
	E89-O···X64-N03-H2	2.77	154.55	31.73
X64-CDK6	V101-O···X64-N02-H1	2.84	160.95	88.23
	V101-N-H···X64-N01	3.14	156.70	85.84
	D104-N-H···X64-N04	3.17	160.39	33.46
	T107-OG1···X64-N04-H4	3.02	127.77	30.01
X3A-CDK2	L91-O···X3A-N15-H7	2.90	151.89	98.63
	L91-N-H···X3A-N1	3.18	159.86	94.38
	E89-O···X3A-N3-H1	2.83	148.07	51.88
	E89-O···X3A-N3-H2	2.82	148.90	46.68
	N140-ND2-HD22···X3A-N8	3.12	153.30	31.04
X3A-CDK6	V101-O···X3A-N15-H7	2.89	162.51	30.34
4AU-CDK2	L91-N-H···4AU-O16	2.93	159.87	96.90
	E89-O···4AU-N7-H5	3.10	157.77	90.72
	L91-O···4AU-N9-H6	2.87	155.87	89.74
4AU-CDK6	V101-N-H···4AU-O16	2.94	151.77	85.50
	E99-O···4AU-N7-H5	2.93	151.36	57.46
	V101-O···4AU-N9-H6	2.91	146.70	56.20

^a^Hydrogen bonds are determined by the acceptor-donor atom distance of <3.5 Å and acceptor-H-donor angle of >120°.

^b^Occupancy (%) is defined as the percentage of simulation time that a specific hydrogen bond exists.

^c^The full lines represent chemical bonds, and the dotted lines indicate hydrogen bonding interactions.

The X64-bound CDK2 *versus* the X64-bound CDK6: X64 yields favourable interactions stronger than 0.9 kcal/mol with I18, V26, A39, E89, F90, L91, Q93, L142, and A152 in CDK2 (Figure 8(A)). The interaction strength of X64 with F90 is scaled by −1.77 kcal/mol and it structurally stems from the π−π interaction of the hydrophobic ring in F90 with that in X64. The interaction energies of X64 with I18, V26, A39, E89, L91, Q93, L142, and A152 are −2.48, −1.85, −1.25, −2.02, −2.78, −1.35, −2.14, and −1.06 kcal/mol, separately, and they are contributed by the CH-π interactions of the alkyls in these eight residues with the hydrophobic ring from X64 (Figure 9(A)). The corresponding frequency distribution of the distance betweenX64 and key residues of CDK2 is depicted in Figure 9(B), which verify that the aforementioned π−π  and CH−π interactions are stable. According to Table 2 and Figure 10(A,B), L91 and E89 in CDK2 form four hydrogen bonding interactions, namely, L91-O···X64-N02-H1, L91-N-H···X64-N01, E89-O···X64-N03-H3, and E89-O···X64-N03-H2 with occupancies of 99.54%, 81.77%, 67.80%, and 31.73%, separately. On the whole, L91 and E89 contribute the interaction energy of −2.78 and −2.02 kcal/mol to the association of X64 with CDK2 (Figures 8(A) and 9(A)). Compared to the X64-bound CDK2, the interaction mode of X64 with CDK6 is similar to those of X64 with CDK2 (Figures 8(B), S5(A), S5(B), S6(A), and S6(B)). By comparison, it is found that the interaction difference of X64 with several common residues (I18, I19), (V26, V27), (A39, V40), (L40, A41), (P53, M54), (E89, R90), (F90, E91), (L91, T92), (Q93, L94), (M99, H100), (D100, V101), (S102, Q103), (L142, H143), (L151, L152), and (A152, V153) in (CDK2, CDK6) is bigger than 0.37 kcal/mol, implying that these residues provide primary contributions for binding selectivity of X64 to CDK2 over CDK6.

The X3A-bound CDK2 over the X3A-bound CDK6: favourable interactions stronger than −0.9 kcal/mol are identified between X3A and seven residues I18, V26, E89, F90, L91, Q93, and L142 in CDK2 (Figure 8(C)). The interaction energy of F90 in CDK2 with X3A is −2.05 kcal/mol, which structurally agrees with the π−π interaction between the hydrophobic ring of X3A and the one of F90 (Figures 8(C) and 9(C)). As exhibited in geometric positions (Figure 9(C)), the CH groups of I18, V26, E89, L91, Q93, and L142 from CDK2 are situated near the ring of X3A, therefore these six residues in CDK2 are easy to produce the CH-π interactions with X3A. Figure 8(C) indicates that I18, V26, E89, L91, Q93, and L142 respectively provide interaction energies of −1.74, −1.55, −1.68, −2.63, −1.10, and −2.10 kcal/mol for the X3A-CDK2 binding. Moreover, X3A forms five HBIs with CDK2, including L91-O···X3A-N15-H7, L91-N-H···X3A-N1, E89-O···X3A-N3-H1, E89-O···X3A-N3-H2, and N140-ND2-HD22···X3A-N8, and their occupancies are 98.63%, 94.38%, 51.88%, 46.68%, and 31.04%, respectively (Table 2 and Figure 10(C,D)). According to Figure 8(D), S4D, S5C, S5D, S6C, S6D, and Table 2, the interactions of X3Awith CDK6 only involves three hydrophobic interactions with energy bigger than 0.9 kcal/mol and a HBI. The interaction difference of X3A with residues (I18, I19), (V26, V27), (K28, K29), (P53, M54), (E89, R90), (F90, E91), (L91, T92), (Q93, L94), and (L142, H143) in (CDK2, CDK6) is stronger than 0.53 kcal/mol, indicating that these residues are primarily responsible for binding selectivity of X3A to CDK2 and CDK6.

The 4AU-bound CDK2 against the 4 AU-bound CDK6: five residues I18, E89, F90, L91, and L142 in CDK2 with 4 AU are −1.59, −1.0, −2.4, −2.32, and −1.56 kcal/mol, respectively, and they structurally arise from the π−π interaction of the hydrophobic ring from F90 with that from 4 AU and the CH-π interactions of the alkyls in I18, E89, L91, and L142 with the hydrophobic ring in 4AU (Figures 8(E) and 9(E,F)). Meanwhile, 4AU forms three HBIs with CDK2, containing L91-N-H···4AU-O16, E89-O···4AU-N7-H5, and L91-O···4AU-N9-H6 with the occupancies are 96.90%, 90.72% and 89.74%, respectively. According to Figure 8(F), S4F, S5E, S5F, S6E, S6F, and Table 2, the binding mode of 4 AU to CDK6 is highly similar to that of 4 AU to CDK2. The interaction difference of 4AU with residues (I18, I19), (E89, R90), (F90, E91), (L91, T92), (M99, H100), (D100, V101), (L142, H143) and (L151, L152) in (CDK2, CDK6) is larger than 1.16 kcal/mol, suggesting that these eight residues contribute key forces to binding selectivity of X3A to CDK2 and CDK6.

## Conclusion

Insights into binding selectivity of inhibitors to CDK2 and CDK6 play a vital role in drug design towards treatment of multiple diseases. This work aims at investigating molecular mechanisms affecting binding selectivity of inhibitors towards CDK2 and CDK6. MSMD simulations of 1.2 μs, consisting of three separate replicas of 400 ns, are implemented on six inhibitor-bound CDK2 and CDK6 systems to decipher selective bindings of X64, X3A, and 4AU to CDK2 and CDK6. The results reveal that the structural flexibility of CDK6 is entirely higher than that of CDK2 and two proteins display diverse internal dynamics behaviour. In addition, the MSA of CDK6 is also larger than that of CDK2, indicating that global structural flexibility of CDK6 is stronger than that of CDK2. The internal dynamics of CDK2 and CDK6 are probed by calculations of DCCMs and PCA, and the results indicate that the bindings of X64, X3A, and 4AU produce enormous influences on the motion modes of CDKs. BFEs of X64, X3A, and 4AU to CDK2 and CDK6 computed by MM-GBSA approach demonstrate that although the binding entropy of X64, X3A, and 4AU to CDK2 is increased compared with that of three inhibitors to CDK6, the increase in the binding enthalpy of X64, X3A, and 4AU with CDK2 relative to that of inhibitors to CDK6 completely screen the increase in unfavourable binding entropy. Hence the compensation between binding enthalpy and entropy plays a key role in binding selectivity of X64, X3A, and 4AU to CDK2 and CDK6. The results obtained from the residue-based free energy decomposition method reveal that five common residues, namely, (I18, I19), (E89, R90), (F90, E91), (L91, T92), and (L142, H143) in (CDK2, CDK6), produce significant differences in bindings of X64, X3A, and 4AU to CDK2 and CDK6, implying that these residues can be regard as efficient targets of designing selective inhibitors towards CDK2 and CDK6. This study is also expected to provide meaningful dynamics information for structure-based design of highly selective inhibitors targeting CDK2 and CDK6.

## Supplementary Material

Supplemental Material

## Data Availability

Supporting data are supplied with the manuscript.
