# Supplementary material for: Binding selectivity-dependent molecular mechanism of inhibitors towards CDK2 and CDK6 investigated by multiple short molecular dynamics and free energy landscapes
Source: J Enzyme Inhib Med Chem. 2022 Nov 7;38(1):84–99. doi: 10.1080/14756366.2022.2135511 (PMC9645286; doi:10.1080/14756366.2022.2135511)
Supplement: Supplemental Material [file IENZ_A_2135511_SM5956.pdf]

Supporting Information

Binding selectivity-dependent molecular mechanism of inhibitors toward CDK2 and CDK6 investigated by multiple short molecular dynamics and free energy landscapes

Lifei Wang[a*], Dan Lu[b],Yan Wang[a], Xiaoyan Xu[a], Peihua Zhong[c] ,and Zhiyong Yang[b*]

[a]School of Science, Shandong Jiaotong University, Jinan, China; [b]Department of Physics, Jiangxi Agricultural University, Nanchang, China; [c]College of Computer Information and Engineering, Jiangxi Agriculture University, Nanchang, China

[*]Corresponding authors. Email: fisherwang@126.com;wanglf@sdjtu.edu.cn (L. Wang), zhiyongyang2009@163.com (Z. Yang).

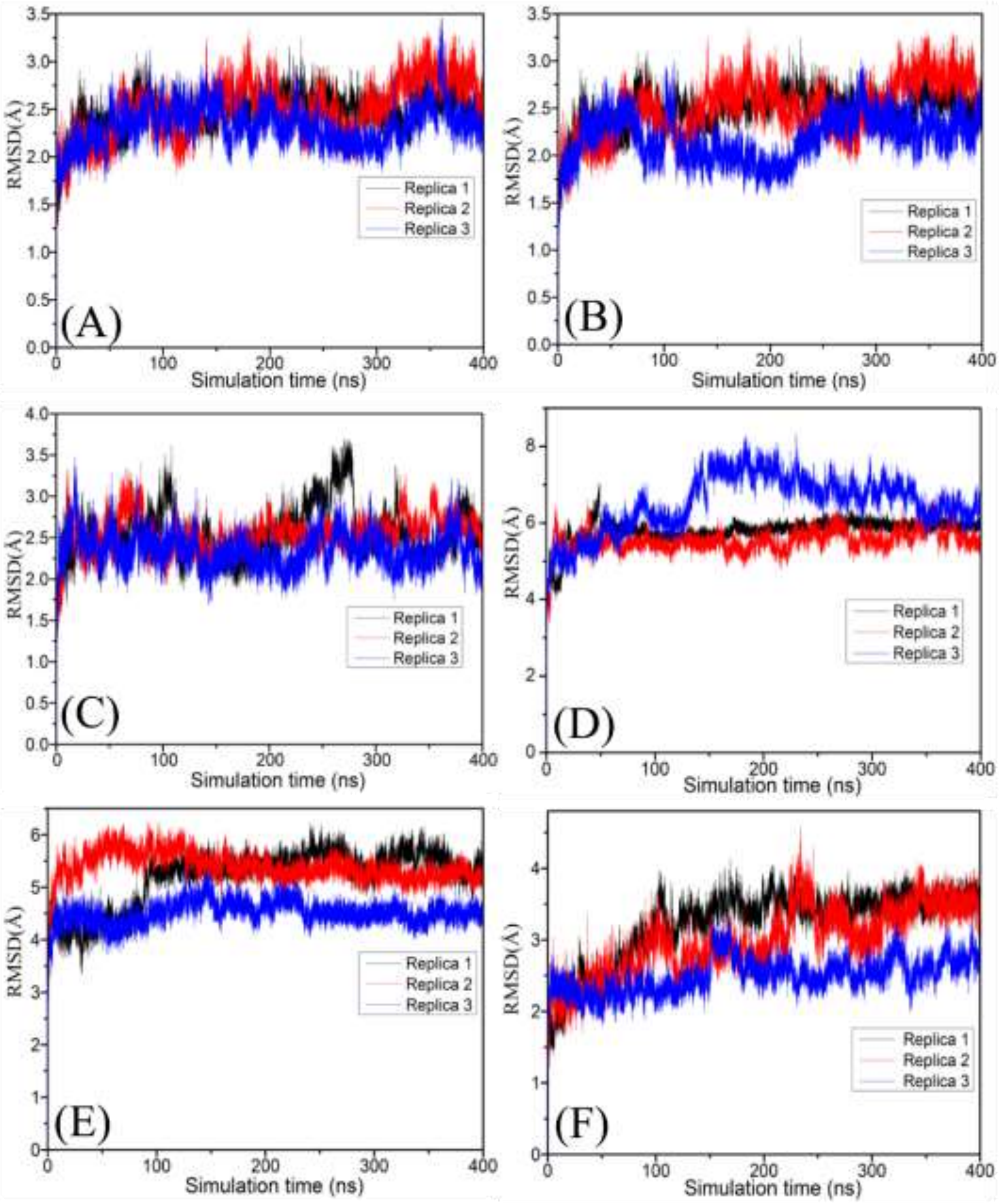

Figure S1. Root-mean-square deviations (RMSDs) of backbone atoms in CDK2 and CDK6 computed by using the SIT: (A) the X64-CDK2 complex, (B) the X64-CDK6 complex, (C) the X3A-CDK2 complex, (D) the X3A-CDK6 complex, (E) the 4AU-CDK2 complex and (F) the 4AU-CDK6 complex.

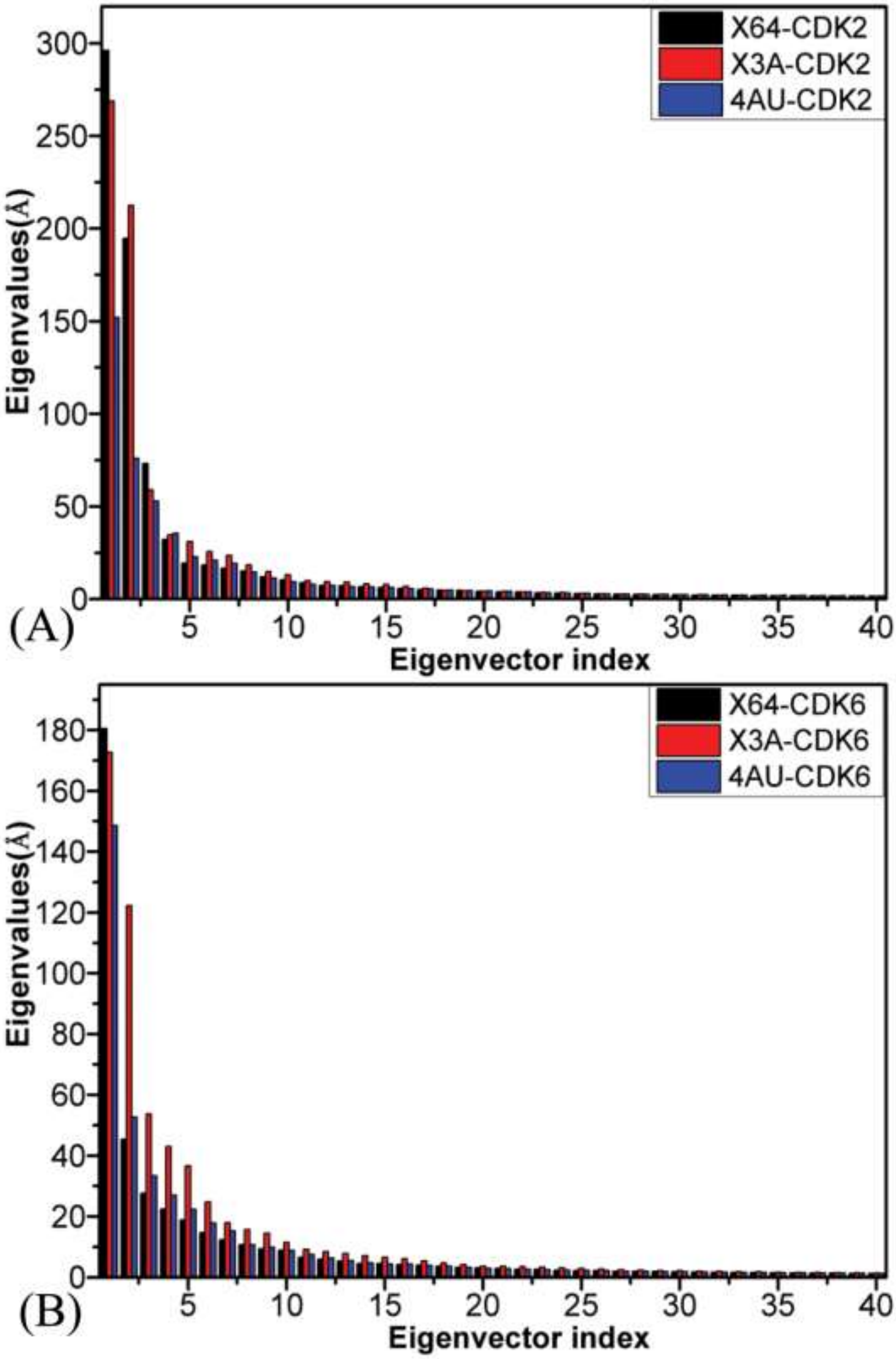

Figure S2. The function of eigenvalues versus eigenvector index stemming from PCA on the SIT: (A) CDK2 and (B) CDK6 complexed with three inhibitors X64, X3A, and 4AU.

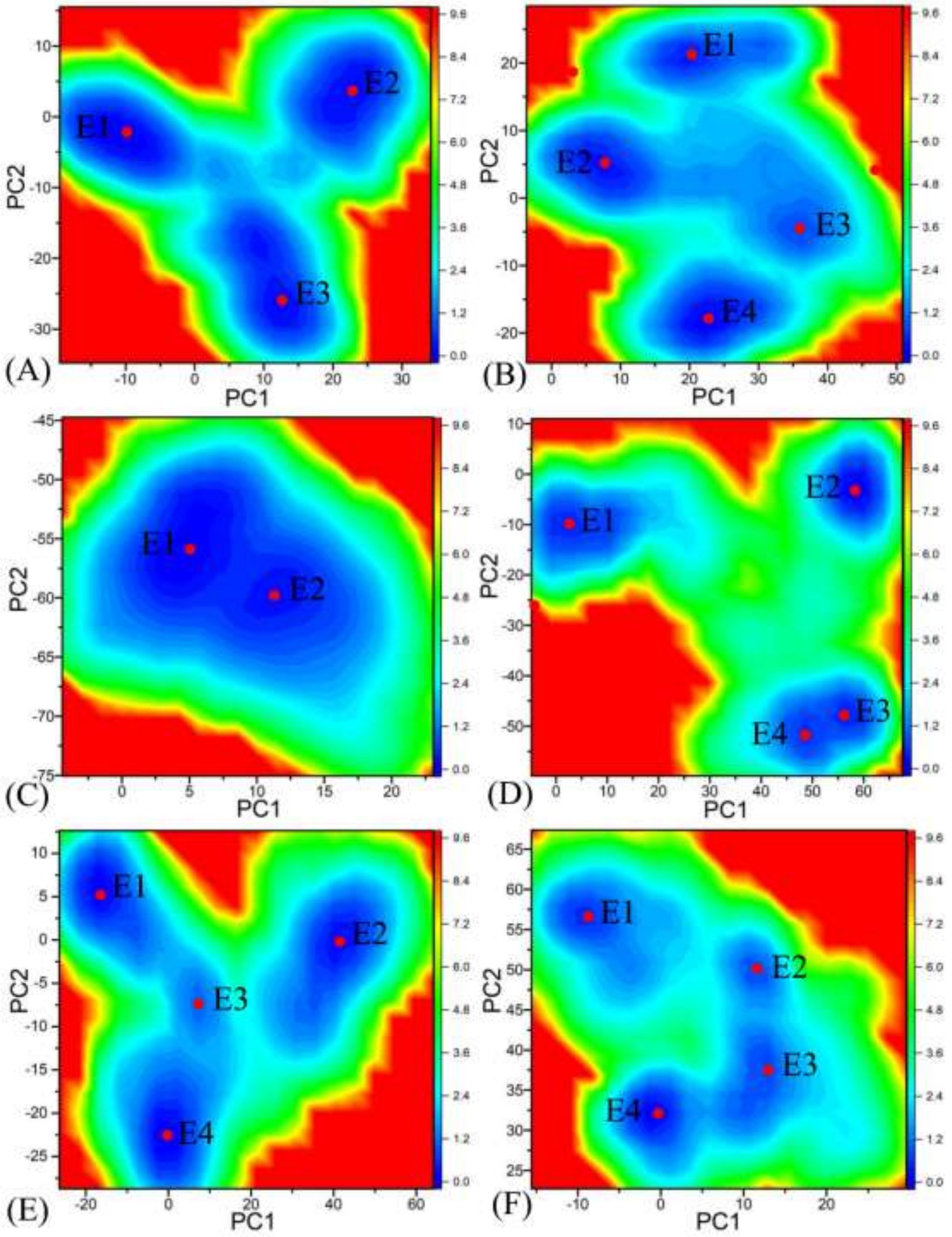

Figure S3. Free energy landscapes constructed by using projections of the SIT onto the first two principal components PC1 and PC2 from the diagonalization of covariance matrix: (A) the X64-CDK2 complex, (B) the X64-CDK6 complex, (C) the X3A-CDK2 complex, (D) the X3A-CDK6 complex, (E) the 4AU-CDK2 complex and (F) the 4AU-CDK6 complex.

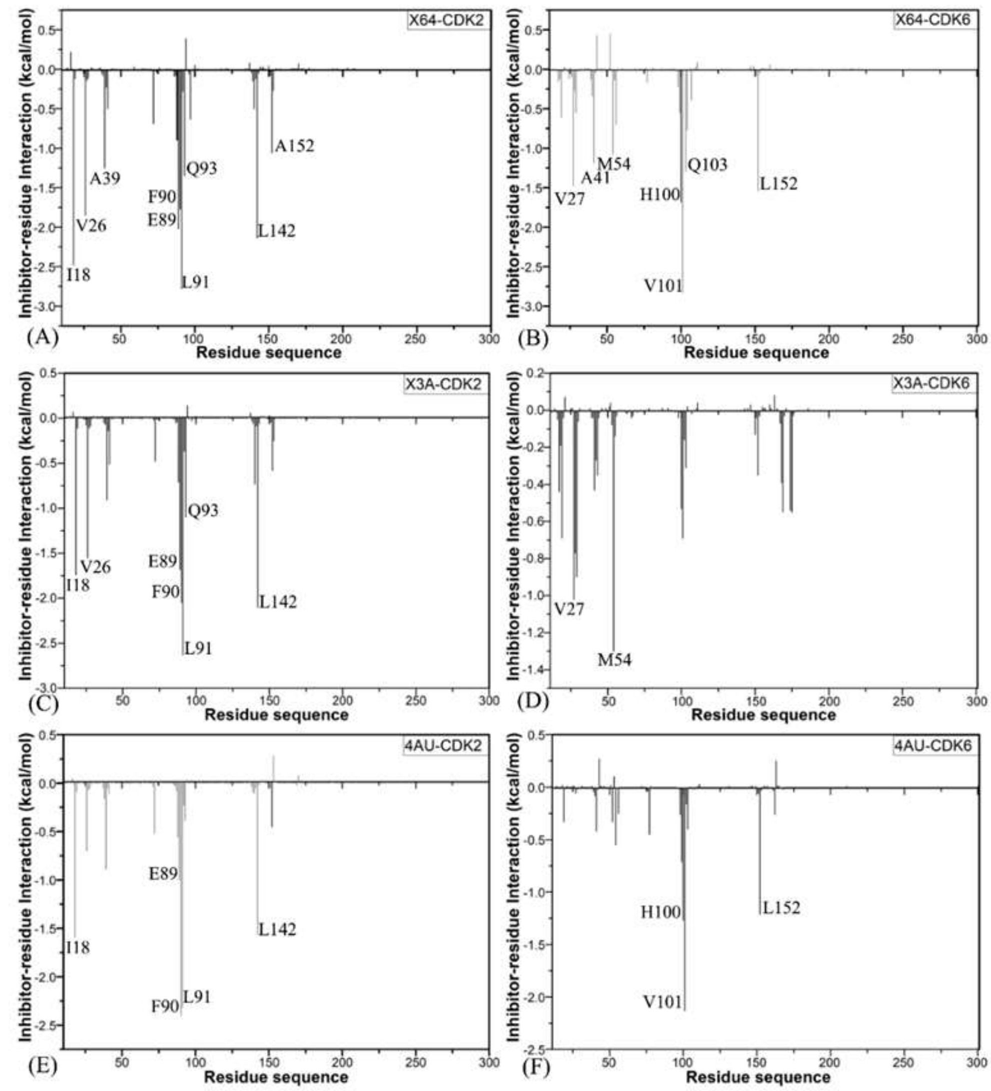

Figure S4. Inhibitor-residue interactions computed by using residue-based free energy decomposition method, only residues stronger than 0.9 kcal/mol are listed: (A) the X64-CDK2 complex, (B) the X64-CDK6 complex, (C) the X3A-CDK2 complex, (D) the X3A-CDK6 complex, (E) the 4AU-CDK2 complex and (F) the 4AU-CDK6 complex.

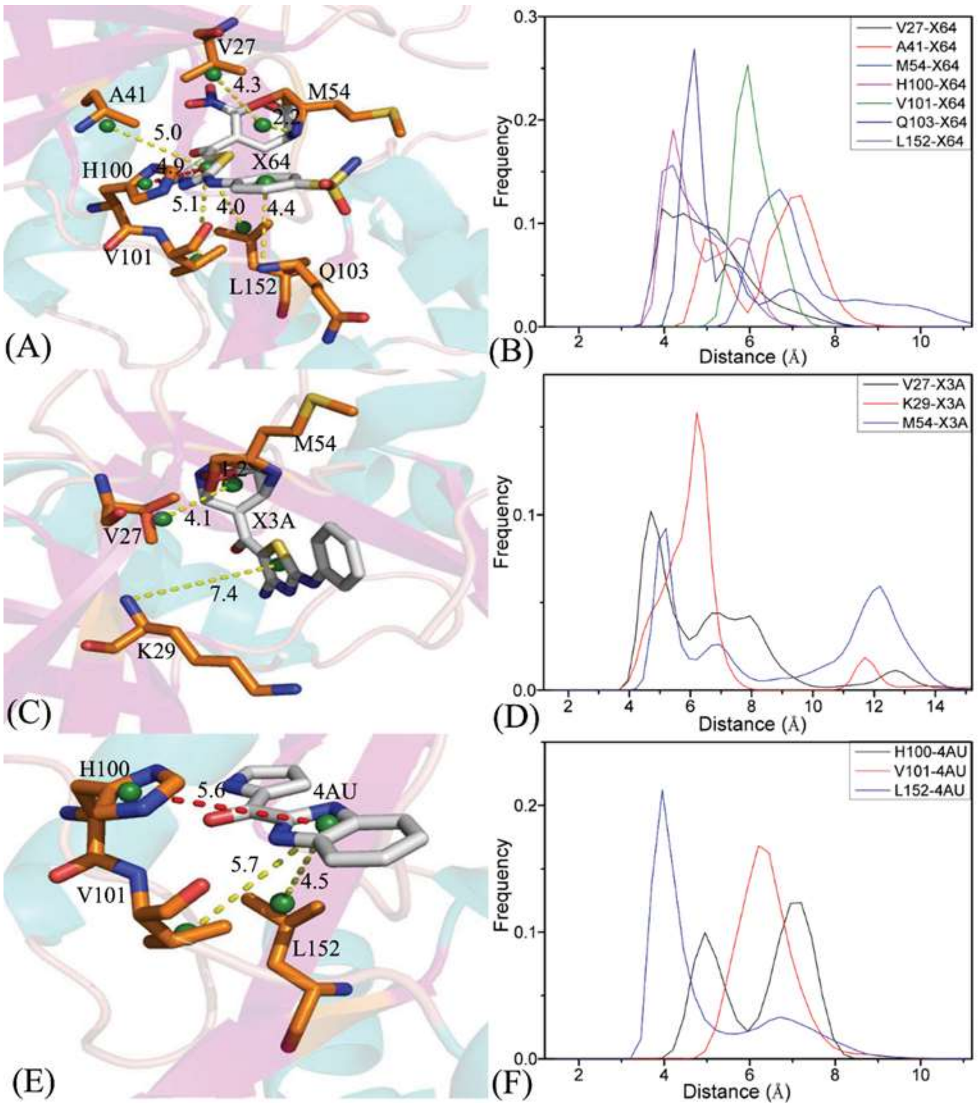

Figure S5. Hydrophobic interactions and the frequency distribution of distance between the inhibitors and key residues of CDK6: (A) the X64-CDK6 complex; (B) RDF of X64-CDK6; (C) the X3A-CDK6 complex; (D) RDF of X3A-CDK6; (E) the 4AU-CDK6 complex; (F) RDF of 4AU-CDK6. The frequency of distances between atoms involving significant interactions were calculated by using the integrated MSMD trajectories of the last 900 ns. The yellow dash lines describe the CH-π interactions and the red dash ones indicate the π-π interactions.

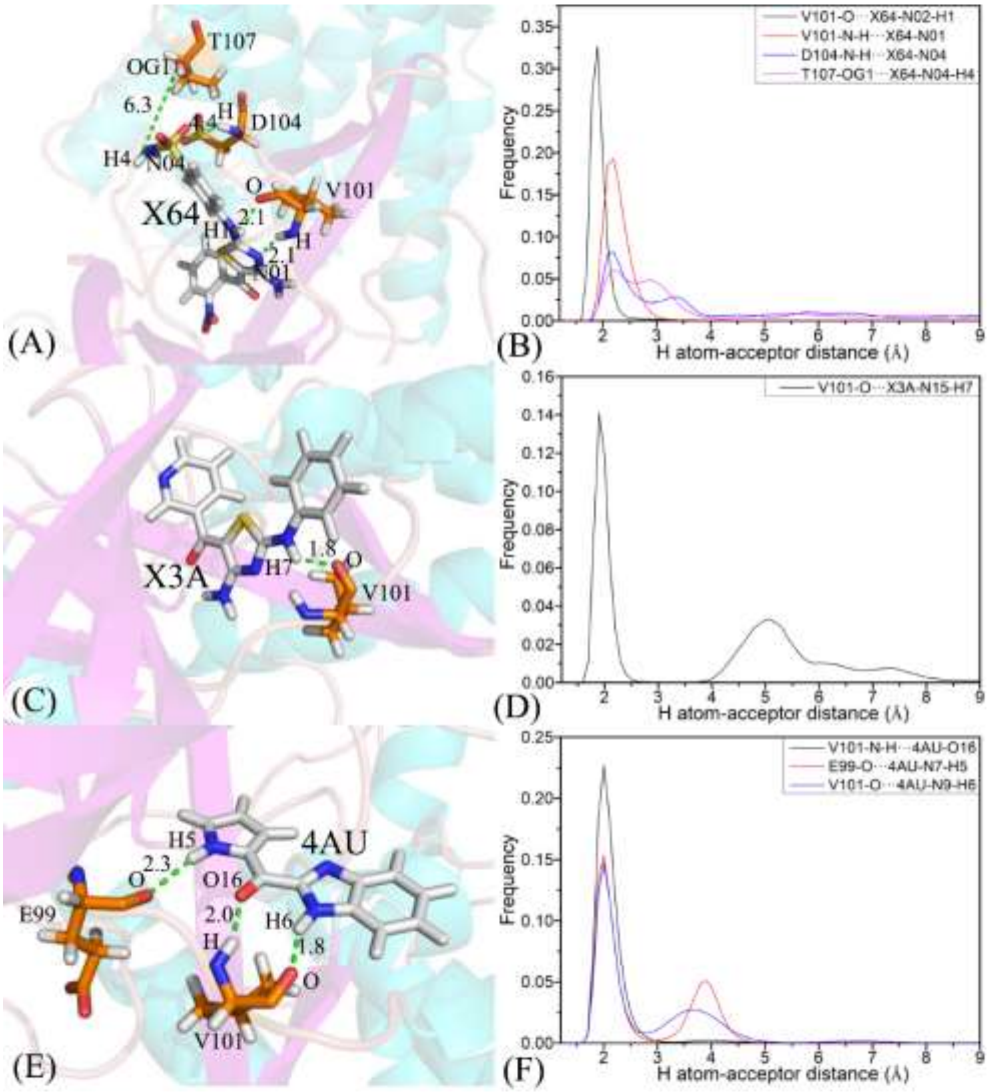

Figure S6. Hydrogen bonds and the corresponding radial distribution function (RDF) of H-O distance between three inhibitors and key residues of CDK6: (A) the X64-CDK6 complex, (B) RDF of H-O distance between V101-O and X64-N02-H1, V101-N-H and X64-N01, D104-N-H and X64-N04, and T107-OG1 and X64-N04-H4; (C) the X3A-CDK6 complex, (D) RDF of H-O distance between V101-O and X3A-N15-H7, (E) the 4AU-CDK6 complex and (F) RDF of H-O distances between V101-N-H and 4AU-O16, E89-O and 4AU-N7-H5, and V101-O and 4AU-N9-H6.
